# Teaching open and reproducible scholarship: a critical review of the evidence base for current pedagogical methods and their outcomes

**DOI:** 10.1098/rsos.221255

**Published:** 2023-05-17

**Authors:** Madeleine Pownall, Flávio Azevedo, Laura M. König, Hannah R. Slack, Thomas Rhys Evans, Zoe Flack, Sandra Grinschgl, Mahmoud M. Elsherif, Katie A. Gilligan-Lee, Catia M. F. de Oliveira, Biljana Gjoneska, Tamara Kalandadze, Katherine Button, Sarah Ashcroft-Jones, Jenny Terry, Nihan Albayrak-Aydemir, Filip Děchtěrenko, Shilaan Alzahawi, Bradley J. Baker, Merle-Marie Pittelkow, Lydia Riedl, Kathleen Schmidt, Charlotte R. Pennington, John J. Shaw, Timo Lüke, Matthew C. Makel, Helena Hartmann, Mirela Zaneva, Daniel Walker, Steven Verheyen, Daniel Cox, Jennifer Mattschey, Tom Gallagher-Mitchell, Peter Branney, Yanna Weisberg, Kamil Izydorczak, Ali H. Al-Hoorie, Ann-Marie Creaven, Suzanne L. K. Stewart, Kai Krautter, Karen Matvienko-Sikar, Samuel J. Westwood, Patrícia Arriaga, Meng Liu, Myriam A. Baum, Tobias Wingen, Robert M. Ross, Aoife O'Mahony, Agata Bochynska, Michelle Jamieson, Myrthe Vel Tromp, Siu Kit Yeung, Martin R. Vasilev, Amélie Gourdon-Kanhukamwe, Leticia Micheli, Markus Konkol, David Moreau, James E. Bartlett, Kait Clark, Gwen Brekelmans, Theofilos Gkinopoulos, Samantha L. Tyler, Jan Philipp Röer, Zlatomira G. Ilchovska, Christopher R. Madan, Olly Robertson, Bethan J. Iley, Samuel Guay, Martina Sladekova, Shanu Sadhwani, FORRT

**Affiliations:** ^1^ School of Psychology, University of Leeds, Leeds LS2 9JT, UK; ^2^ Department of Psychology, University of Cambridge, CB2 3EB, UK; ^3^ Faculty of Life Sciences: Food, Nutrition and Health, University of Bayreuth, 95447 Bayreuth, Germany; ^4^ School of Psychology, University of Nottingham, Nottingham NG7 2RD, UK; ^5^ School of Human Sciences, University of Greenwich, London SE10 9LS, UK; ^6^ Centre for Workforce Development, Institute for Lifecourse Development, University of Greenwich, London SE10 9LS, UK; ^7^ School of Humanities and Social Science, University of Brighton, BN2 0JY, UK; ^8^ Institute of Psychology, University of Graz, 8010 Graz, Austria; ^9^ School of Psychology, University of Birmingham, B15 2TT, UK; ^10^ School of Psychology, University College Dublin, Dublin 4, DO4 V1WD, Ireland; ^11^ Department of Psychology, University of York, York YO10 5DD, USA; ^12^ Macedonian Academy of Sciences and Arts, North Macedonia, XCWR+GJM, 1000; ^13^ Faculty of Teacher Education and Languages, Department of Education, ICT and Learning, Ostfold University College, 1757 Halden, Norway; ^14^ Department of Psychology, University of Bath, Bath BA2 7AY, UK; ^15^ Department of Experimental Psychology, University of Oxford, Oxford OX1 4BH18, UK; ^16^ School of Psychology, University of Sussex, Brighton BN1 9RH, UK; ^17^ School of Psychology and Counselling, the Open University, Milton Keynes MK7 6AA, UK; ^18^ Department of Psychological and Behavioural Science, London School of Economics and Political Science, UK; ^19^ Department of Mathematics, College of Polytechnics Jihlava, 1556/16, 586 01, Czech Republic; ^20^ Graduate School of Business, Stanford University, CA 94305, USA; ^21^ Department of Sport and Recreation Management, Temple University, PA 19122, USA; ^22^ Department of Psychology, University of Groningen, 9712 CP, Groningen, the Netherlands; ^23^ Department of Psychiatry and Psychotherapy, Philipps-University Marburg, D-35039 Marburg, Germany; ^24^ Department of Psychology, Ashland University, OH 44805, USA; ^25^ School of Psychology, Aston University, Birmingham B4 7ET, UK; ^26^ Division of Psychology, De Montfort University, Leicester LE1 9BH, UK; ^27^ Institute for Educational Research and Teacher Education, University of Graz, Graz, 8010 Graz, Austria; ^28^ School of Education, Johns Hopkins University, MD 21218, USA; ^29^ Department for Cognition, Emotion, and Methods in Psychology, University of Vienna, Vienna 1010, Austria; ^30^ Department of Psychology, Education and Child Studies, Erasmus University Rotterdam, Rotterdam 3000, The Netherlands; ^31^ Division of Neuroscience and Experimental Psychology, University of Manchester, Manchester M13 9PL, UK; ^32^ Department of Psychology, Liverpool Hope University, Liverpool L16 9JD, UK; ^33^ Department of Psychology, Faculty of Management, Law and Social Sciences, University of Bradford, Bradford BD7 1DP, UK; ^34^ Department of Psychology, Linfield University, Linfield, 503-883-2200, USA; ^35^ Faculty of Psychology in Wrocław, SWPS University of Social Sciences and Humanities, Wrocław 03-81536, Al Jubail 35819, Poland; ^36^ Jubail English Language and Preparatory Year Institute, Royal Commission for Jubail and Yanbu, Saudi Arabia; ^37^ Department of Psychology, University of Limerick, V94 T9PX, Ireland; ^38^ School of Psychology, University of Chester, Chester CH1 4BJ, UK; ^39^ Department of Psychology, Saarland University, 66123 Saarbrücken, Germany; ^40^ School of Public Health, University College Cork, Cork, T12 K8AF, Ireland; ^41^ Department of Psychology, School of Social Science, University of Westminster, London W1B 2HW, UK; ^42^ Iscte-Universty Institute of Lisbon, CIS-IUL, 1649-026 Lisboa, Portugal; ^43^ Faculty of Education, University of Cambridge, Cambridge CB2 1TN, UK; ^44^ Institute of General Practice and Family Medicine, University Hospital Bonn, University of Bonn, 53127 Bonn, Germany; ^45^ Department of Philosophy, Macquarie University, NSW 2109, Australia; ^46^ School of Psychology, Cardiff University, Cardiff CF10 3AT, UK; ^47^ University Library, University of Oslo, 0313 Oslo, Norway; ^48^ School of Social and Political Sciences, University of Glasgow, Glasgow G12 8QQ, UK; ^49^ School of Psychology and Neuroscience, University of Glasgow, Glasgow G12 8QQ, UK; ^50^ Department of Psychology, Leiden University, 2311 EZ Leiden, The Netherlands; ^51^ Department of Psychology, the Chinese University of Hong Kong, Hong Kong, SAR 100871, People's Republic of China; ^52^ Department of Psychology, Bournemouth University, Poole BH12 5BB, UK; ^53^ Department of Neuroimaging, IoPPN, King's College London, UK; ^54^ IGDORE, London, UK; ^55^ Department of Psychology III, University of Würzburg, 97070 Würzburg, Germany; ^56^ Faculty for Geo-Information Science and Earth Observation, University of Twente, 7522 NB, The Netherlands; ^57^ School of Psychology, University of Auckland, Auckland 1142, New Zealand; ^58^ Department of Social Sciences, University of the West of England, Bristol BS16 1QY, UK; ^59^ Department of Biological and Experimental Psychology, Queen Mary University of London, E1 4NS, UK; ^60^ South-East European Research Centre, Thessaloniki, Greece; ^61^ Department of Neuroscience, Psychology and Behaviour, University of Leicester, UK; ^62^ Department of Psychology, Witten/Herdecke University, Germany; ^63^ Departments of Psychiatry and Experimental Psychology, University of Oxford, UK; ^64^ School of Psychology, Keele University, Newcastle ST5 5BG, UK; ^65^ School of Psychology, Queen's University Belfast, Belfast BT7 1NN, UK; ^66^ Department of Psychology, University of Montreal, Canada; ^67^ Framework for Open and Reproducible Research Training

**Keywords:** higher education, open research, open scholarship, open science, pedagogy, teaching

## Abstract

In recent years, the scientific community has called for improvements in the credibility, robustness and reproducibility of research, characterized by increased interest and promotion of open and transparent research practices. While progress has been positive, there is a lack of consideration about how this approach can be embedded into undergraduate and postgraduate research training. Specifically, a critical overview of the literature which investigates how integrating open and reproducible science may influence *student outcomes* is needed. In this paper, we provide the first critical review of literature surrounding the integration of open and reproducible scholarship into teaching and learning and its associated outcomes in students. Our review highlighted how embedding open and reproducible scholarship appears to be associated with (i) students' *scientific literacies* (i.e. students’ understanding of open research, consumption of science and the development of transferable skills); (ii) *student engagement* (i.e. motivation and engagement with learning, collaboration and engagement in open research) and (iii) students' *attitudes towards science* (i.e. trust in science and confidence in research findings). However, our review also identified a need for more robust and rigorous methods within pedagogical research, including more interventional and experimental evaluations of teaching practice. We discuss implications for teaching and learning scholarship.

## 1. Teaching open and reproducible scholarship: a critical review of the evidence base for current pedagogical methods and their outcomes

In response to concerns surrounding the credibility, robustness and transparency of research, there has been a noticeable acceleration in the adoption of open and reproducible scholarship tools [1,2]. Open scholarship^1^ broadly refers to a set of principles and practices that aim to increase the transparency, rigour, reproducibility, replicability, incrementalism and inclusivity of research [4]. Many tools have been developed to facilitate these goals, such as study pre-registration and Registered Reports (e.g. [6–8]), open materials, code and/or data [9], open access publishing [10] and a focus on replication studies [11–14]. The usefulness of such tools is not confined to the social sciences and they have also been considered across other disciplines (e.g. animal behaviour [15,16], cancer biology [17], and economics [18]). While the discussions surrounding openness and reproducibility have led to promising and productive changes in research culture (e.g. [19–21]), there remains progress to be made (see [22–24]). Specifically, there is now an urgent, need to embed the principles of open scholarship into teaching, learning, training and pedagogy [1,2]. Indeed, if open and reproducible scholarship is not explicitly embedded into undergraduate and postgraduate teaching and learning, students will not be well equipped with the knowledge and skills to continue advancing these goals and thus will not be able to contribute to sustained culture change [3,25]. It is, therefore, crucial that the pedagogical aspect of open and reproducible scholarship is at the centre of ongoing discussions.

Many scholars have made the ‘moral case’ for adopting open scholarship tools, noting how open scholarship is necessary for the accessibility, inclusivity, robustness and advancement of research (e.g. [26–29]). While there have been some useful discussions surrounding the need for open scholarship in teaching and learning contexts in recent literature (e.g. [30,31]), a comprehensive summary of the *empirical* evidence is lacking.

### 1.1. Aim

The aim of this paper is to review and synthesize evidence that explores embedding open and reproducible scholarship in teaching and learning contexts. In our review, we synthesized the empirical evidence that investigates whether open scholarship influences students' scientific literacy, engagement and attitudes towards science. When we evaluated the evidence which explores the integration of open and reproducible scholarship into teaching, we considered a diverse range of practices. For example, we were interested in approaches to teaching students *about* replicability, open scholarship, pre-registration and other approaches, and generally implementing such topics in the curriculum. Similarly, we also reviewed the *impact* of allowing students to experience first-hand open scholarship through, for example, using open resources, open data and hands-on research experience. In addition, part of our goal is to advocate for a more encompassing involvement of open scholarship approaches into teaching, which could allow students to experience them in practice, in order to judge their benefits. This broader and more encompassing view on open scholarship practices is also included in the term we use itself. To this end, we define open and reproducible scholarship as a set of practices which endeavour to make scientific research, knowledge and empirical data (and dissemination thereof) widely accessible, rigorous and available to professionals and citizens. Indeed, scientific progress becomes feasible and the scientific process more rigorous, transparent and reproducible [4,32,33]. Our review responds to the key components of definition of open and reproducible science by synthesizing literature which summarizes how teaching open and reproducible scholarship may be associated with outcomes including (i) scientific literacy, (ii) engagement and (iii) attitudes towards research. These themes represent three fundamental aspects of the student experience that capture both students’ development of skills *during* teaching and learning and down-stream consequences following their research training.

## 2. Literature search strategy

This paper presents a synthesis of the evidence relating to the teaching of open and reproducible scholarship, including a review of related student outcomes. Here, we informed a narrative evidence synthesis with a rigorous systematic review in order to: (i) include the evidence that exists in non-traditional spaces (e.g. grey literature, including unpublished student evaluations and online talks); (ii) ensure that the full breadth of definitions of open and reproducible scholarship was captured and (iii) rigorously evaluate the evidence in this area. This review includes studies involving undergraduate and postgraduate students, in all subject areas, on a global level.

### 2.1. Establishing the search strategy

To identify potentially relevant studies, we initially searched a wide evidence base with a targeted research question: what are the impacts of incorporating open and reproducible principles into teaching upon student students' scientific literacies, student engagement and attitudes towards science? At the start of this project, we were initially interested in understanding how open scholarship may impact tangible outcomes in students, and we anticipated that the literature would allow us to investigate the direct causal consequences of open scholarship. However, given the state of the evidence and the lack of experimental studies that directly measured impact specifically, we since readdressed this research question to instead summarize literature which explores the broader outcomes that are *associated with* open and reproducible scholarship. We aimed to conduct an unbiased and impartial search, so we created an *a priori* strategy containing search terms, search places and inclusion criteria. The search strategy was devised by a subset of the authorship team with expertise in literature reviews (see CRedIT Statement; https://osf.io/hg7nt/). This search strategy was informed by guidelines for best practice in non-intervention reviews, including NIRO [34] and SPIDER (table 1) and helped us identify only empirical papers, including (un)published works. The search included main academic platforms for formally published research (Web of Science, Scopus, EBSCOhost, Pubmed, Medline, Embase), preprint archives (PsyArXiv; ProQuest dissertations, Open Science Framework, EdArXiv, MetaArXiv), as well as additional academic resources for emerging and unpublished research (Education Resources Information Center [ERIC], Bielefeld Academic Search Engine, OpenGrey), the FORRT community and Twitter. The search strategy in full, including search places, search terms and results can be accessed here: https://osf.io/29qvh.

**Table 1.** Outline of search strategy, as informed by SPIDER [35].

SPIDER category	strategy in the present paper
sample	university students; higher education; undergraduate
phenomenon of interest	replication; open science; open scholarship; open research; open practices; open principles; open pedagogy; pre-registration; registered reports; ‘reproducib*’; reproducible; reproducible science; crowdsourcing; "*registr’
design	qualitative, quantitative, or mixed-methods
evaluation	attitudes OR student engagement OR perception OR outcomes
research type	qualitative and mixed-methods; experimental; observational

The complete inclusion criteria were as follows:

1. The paper discusses open and reproducible scholarship in the context of Higher Education, including (but not limited to) undergraduate students, taught postgraduate students and/or research students (e.g. Masters and PhD by Research).
2. The paper specifically mentions open and/or reproducible science/scholarship and discusses student (undergraduate or postgraduate) outcomes.

Search terms included: teaching; mentoring; pedagogy; open scholarship; open educational resources; replication; reproducibility; research repository; student projects; empirical dissertation; team science; data sharing; replication crisis; reproducibility crisis; Many Labs; Hagen Cumulative Science Project; questionable research practices (QRPs); responsible conduct of research; detrimental ethical practice; educational practice; Collaborative Replications and Education Project (CREP); dissertation; College Teaching; Framework of Open Reproducible Research Training (FORRT); Project Teaching Integrity in Empirical Research (TIER); research transparency; service learning; credibility; preprints; registered reports; public understanding of science; science communication; epistemic trust; trust in science; attitudes toward science; open-source materials; undergraduate pre-registration assignment; scholarship of teaching and learning; higher education.

### 2.2. Value of a Team Science approach

This project used a large, interdisciplinary Team Science approach to conduct the review and synthesize the evidence. Team Science approaches are useful for enhancing collaboration and increasing resources [36]. In this vein, we were able to include diverse perspectives due to contributors’ varied academic and cultural backgrounds, which we deemed especially important since approaches to and requirements for teaching in Higher Education vary substantially between countries. Contributors stemmed from academic institutions in 17 countries on four continents and represented a wide range of disciplines including communication science, economics, educational sciences, geoscience, neuroscience, psychology, public health and sports science. Furthermore, this approach allowed us to pool resources such as access to databases, as well as to complete the search and screening in a short time frame, maximizing the use of multiple authors with differing expertise.

#### 2.2.1. Literature search

After devising and finalizing our search strategy (table 1), a sub-team then ran a comprehensive search of the literature; the full protocol can be accessed here: https://osf.io/4jqbw/. The search was conducted from December 2021 to January 2022. This search resulted in an initial selection of 866 identified papers. A second sub-team then reviewed each paper against the two above inclusion criteria, categorized each paper as qualitative/quantitative/mixed methods, noted the student sample (undergraduate or postgraduate) and provided additional comments. Each paper was independently evaluated by two coders for its relevance, who were each naive to one another's decision, using separate tabs on a shared Google Sheet to facilitate collaboration. Coders were randomized to one of two coding sheets and randomization of authors to coding was achieved using author surnames. When a paper was deemed to fit the inclusion criteria, coders assigned it to the three thematic categories (*scientific literacies, student engagement* or *attitudes towards science*). These categories were not mutually exclusive; that is, a paper could be assigned to more than one thematic category.

After coding was complete, entries that did not meet the inclusion criteria were excluded (*N* = 829). A further paper was also excluded because it was removed online by the authors during the review process. See electronic supplementary material for a flow chart of the full screening process (https://osf.io/9xghj). Interrater reliability was strong (*κ* = 0.915). Discrepancies between codes were discussed and resolved with the wider team, leading to a final set of 36 screened papers (see table 2 for an exhaustive account of the design, sample size and summaries of these papers). The 36 papers identified by the systematic review process are indicated with an asterisk in the reference list. The core writing team (see *CRediT statement*) then received a shared Zotero drive which contained all of the relevant screened papers, organized by three distinct categories: *scientific literacies, student engagement* and *attitudes towards science.* Sub-categories were also identified (table 3). To the initial list of 36 papers, we also added to the project shared drive other relevant papers and grey literature, in an iterative manner, following wider review of the literature; these papers provided additional context. This was primarily achieved using forward and backward citation searching of the relevant papers identified by the review, as well as wider searches in grey literature that were not covered by the review; for example, links shared on Twitter, the Project TIER website and the FORRT community. In the review, we identified broadly two different types of research, either (i) research which considers the integration of open and reproducible scholarship tools and practices (which included topics like discussing the replication crisis and QRPs with students; teaching students how to undertake specific open practices, such as pre-registration and conducting replication studies; and discussing with students such practices in the context of evaluating research papers), or (ii) research which evaluates the use of open educational resources (which are often, but are not necessarily, *about* research practices). We now summarize the evidence across the three thematic categories, before discussing wider implications. Note that some papers were identified as relevant across subsections of the review and these are included in all relevant subsections, with more detail in their first mention.

**Table 2.** Summaries of papers identified by the systematic review.

	paper	sample size	sample description (UG/PG etc)	effect size (if relevant)	design	country
1	Afolabi [37]	*n* = 106	university undergraduates	not applicable	between-group pre-post-test design	Nigeria
2	Al Abri & Dabbagh [38]	*n* = 12 (11 graduate students, 1 course instructor)	graduate students and course instructor	not applicable	sequential mixed-method design	USA
3	Altunoglu [39]	five focus groups (each with min. six students)	university students	not applicable	interpretive qualitative case study design	Turkey
4	Anglin & Edlund [30]	*n* = 328	instructors of psychology	for type of course where replication and reform issues are discussed: *η*^2^ = 0.05 for graduate versus advanced undergraduate versus introductory undergraduate courses; *η*^2^ = 0.18 for introductory methods versus general versus content courses; *d* = 0.84 for advanced undergraduate methods versus content courses; *d* = 0.93 for graduate methods versus other courses	single time-point questionnaire study	mainly USA (*n* = 291)
5	Andone, Mihaescu, Vert, Ternauciuc, & Vasiu [40]	*n* = 120	university undergraduates (bachelor students) and graduates (master students)	not applicable	qualitative research design (case studies implementing OER) and interpretation	Timișoara, Romania
6	Baran & AlZoubi [41]	*n* = 13	university undergraduates	not applicable	qualitative design (single case study) and interpretation (via reflection reports and semi-structured interviews)	USA
7	Becker [42]	small intensive class (no precise *n* given)	university undergraduates (upper division)	not applicable	descriptive case study of course content relating to qualitative replication and its implementation	USA
8	Bloom [43]	*n* = 92	university undergraduates (first-year students)	not applicable	Wilcoxon Rank Sum test yielded no statistically significant difference between control (no OER) and treatment (yes OER) group	USA
		(*n*_control_ = 32				
		*n*_treatment_ = 60)				
9	Button *et al.* [44]	not applicable	university undergraduates	not applicable	descriptive commentary reporting student completion numbers, anecdotal evidence, text summary of themes from student feedback forms	UK
10	Çetinkaya-Rundel & Ellison [45]	not applicable	university undergraduates	not applicable	descriptive commentary reporting anecdotal evidence	USA
11	Chopik *et al.* [46]	*n* 194	university undergraduates	*d* −0.36 to 0.21 for comparisons of items measuring attitudes towards Psychology	within-subjects pre- versus post-intervention comparison	USA
12	Davis & Parmenter [47]	*n* = 7 female outreach ambassadors	outreach ambassadors (6 undergraduates, 1 postgraduate)	not applicable	narrative production- qualitative case study design via interviews	UK
13	Hare *et al.* [48]	*n* = 34 An online cohort (*n* = 13) and two on-campus cohorts (*n* = 21)	doctoral students	not applicable	students’ discourses to open-response questions in discussion forum. Thematic analysis, which also quantitatively illustrating the categories that were represented.	USA
14	Jekel *et al.* [49]	*n* = 80 completed theses that are replication studies	university undergraduates	not applicable	descriptive commentary of course content with anecdotal evidence	Germany
15	Krishna & Peter [50]	*n* = 207	individuals who were currently completing or had completed in the last 2 years a Bachelor's or Master's research thesis at a German public university	*d_z_* = 0.43 for engaging in analysis and reporting QRPs vs. design QRPs; *Adj. R^2^* = 0.212 for the model of predictors of analysis and reporting QRPs; *Adj. R*^2^ = 0.016 for the model of predictors of design QRPs; *R*^2^ = 0.185 for the mediation model for analysis and reporting QRPs; *R*^2^ = 0.069 for the mediation model for design QRPs	single time-point self-report questionnaire study	Germany
16	Lin [51]	*n* = 58	university undergraduate students	not applicable	qualitative research (online survey and two focus groups).	USA
17	Marwick *et al.* [52]	*n* = 16 in the class;	university undergraduate students	not applicable	qualitative research (replication assignments for an archeology class and feedback survey).	USA
		*n* = 13 in the evaluation				
18	Marshall & Underwood [53]	not applicable	university undergraduate students	not applicable	description of an empirical research project as a component of an upper-level undergraduate economics writing-in-the-discipline course	USA
19	McCright [54]	experimental group (*n* = 27)	undergraduate students	not applicable	quasi-experimental study about inquiry-based learning project	USA
		control (*n* = 130)				
20	Pan [55]	*n* = 518	graduate students	η2 from 0.02 to 0.13 on difference between Taiwanese and American students understanding of responsible conduct of research	online survey using diagnostic assessment, the Revises RCR [responsible conduct of research] Reasoning Test	Taiwan and USA
21	Paviotti *et al.* [56]	*n* = 24 students	students and staff	not applicable	mixed-methods evaluation, using questionnaires and interviews	Italy
		*n* = 5 staff				
22	Poronnik & Moni [57]	*n* = 230	final-year physiology students	not applicable	Likert-style questionnaires	Australia
23	Pyott [58]	*n* = 19	undergraduate students	not applicable	hands-on learning activity to teach experimental design, reflections on the classes/activities	USA
24	Ryan [59]	*n* = 8	university undergraduates	not applicable	module evaluations and opportunities for students to demonstrate learning outcomes (either via an oral interview or written assessment).	UK
25	Sacco & Brown [60]	*n* = 49	psychology graduate	QRP endorsement significantly decreased from pre- to post-intervention ($\eta_{p}^{2}=0.194$ ); positive attitudes/satisfaction towards the intervention correlated with more learning, *r* = 0.47/ *r* = 0.42	Likert-style questionnaires and intervention	USA
26	Sanchez *et al.* [61]	*n* = 144	students	not applicable	qualitative free-text student feedback	USA
27	Sarafoglou *et al.* [62]	*n* = 43	research master psychology students	not applicable	protocol of a Research Master Course “Good Research Practices”	Netherlands
28	Sawchuk [63]	*n* not specified	undergraduate students	not applicable	exercise for replications in an active-learning classroom (qualitative and quantitative analysis)	Canada
29	Smith *et al.* [64]	*n* = 24 students (2 full, 22 partial replications)	biostatistics PhD students	not applicable	training to conducting full and partial replications	USA
30	Steinhardt [65]	*n* = 13 (9 sociology, 4 teacher training)	bachelor students in sociology and teacher training	not applicable	seminar plan for teaching open science practices in qualitative research	Germany
31	Stiemsma *et al.* [66]	*n* not specified	STEM undergraduate students	not applicable	anecdotal descriptive evaluation of students as scholars programme	USA
32	Tillinghast *et al.* [67]	*n* = 127 students;	undergraduate students	not applicable	mixed-methods	USA
		*n* = 113 in a survey; *n* = 9 in interviews				
33	Toelch & Ostwald [68]	*n* = 17 completed evaluation	mixed graduate students (MSc and PhD)	not applicable	evaluation with open-text comments	Germany
34	Wang *et al.* [69]	Australia (*n* = 14,530) and Taiwan (*n* = 7708)	students	not reported	online study using validated measures	Australia and Taiwan
35	Watson *et al.* [70]	*n* = 1299	undergraduate biology students	not applicable	online survey	USA
36	Werth & Williams [71]	survey *n* = 92	undergraduate students	not applicable	mixed-methods descriptive study of student perceptions	USA
		interviews *n* = 12

**Table 3.** Thematic categories and sub-categories identified by the literature review.

thematic category	sub-categories identified by the literature review
scientific literacies	understanding of open research, students' consumption of science, development of transferable skills
student engagement	motivation and engagement with learning, collaboration and student involvement, engagement in open research behaviours
student attitudes towards science	students' trust in science, confidence in research findings

## 3. Open and reproducible scholarship and students' scientific literacies

The first thematic category that was highlighted by our review and guided by our research question was 'scientific literacies'. Recently, there has been a heightened emphasis on scientific literacies as a core competency for undergraduate and graduate education [72,73]. Scientific literacy refers to the knowledge, skills, competencies and attitudes related to both awareness of and participation in scientific culture and also ‘doing’ science, i.e., engaging in scientific investigations (for a review on how scientific knowledge has been conceptualized see [73,74]). In research-based subjects, scientific literacies may also include statistical competencies, understanding of research practices and other practical research skills students develop throughout their studies. A concern for scientific literacies is becoming rapidly integrated within accreditation and policy in Higher Education across countries (e.g. in the UK, [75], and in the USA, [76,77]). Therefore, it is necessary to reflect on how scientific literacy can be impacted by the adoption of open and reproducible scholarship practices in students' curricula.

Scientific literacies, in the context of the present paper, refer to competencies and skills developed by students during their program, which are generally related to the production and consumption of science and scientific knowledge. Previous work has investigated interventions to improve students’ scientific literacies, for example, with initiatives such as inquiry-based learning projects with students [54] and hands-on science training for school-aged students [69], with positive outcomes. In this context, embedding open and reproducible approaches to research teaching and learning may also aid students' development of discipline-specific scientific literacy [30]. Our review suggests three broad areas within students’ scientific literacy that can be impacted by the implementation of open and reproducible scholarship: (i) students' understanding of open research, (ii) students’ consumption of science and (iii) the development of transferable skills. We now detail each of these in turn.

### 3.1. Students’ understanding of Open Research

Overall, our review showed evidence that explicitly embedding open scholarship tools, for example, study pre-registration (i.e. the process of creating a time-stamped account of study hypotheses, methods and planned analyses before data analysis; [4]), into coursework can help students to be more *literate* with the interpretation of statistical results. For example, Blincoe & Buchert [78] found that undergraduate psychology students (*n* = 36) reported pre-registration to be a helpful planning tool because it allowed them to understand the value that null or non-significant findings have in research. This was measured using a survey after a pre-registration assignment implemented in a research course in a psychology programme. As Blincoe & Buchert [78] explain, pre-registering research with undergraduates can also serve to demonstrate to students how QRPs (i.e. problematic practices that researchers engage in to improve the chances of gaining significant results; [4]) can be reduced, especially with regard to recognizing the difference between *a priori* and *post hoc* research decisions. Thus, the incorporation of pre-registration in pedagogy may promote best practice in quantitative research and strengthen students' understanding of research findings which, in turn, could promote scientific literacies. There was also evidence in our review that implementing study pre-registration may help students develop their scientific literacies in other ways. For example, Pownall [79] recommends that aligning the quantitative analyses more clearly with students’ original research questions through pre-registration may increase the perceived usefulness of statistics.

Our review also highlighted useful case study examples of how embedding open and reproducible scholarship into teaching and learning can improve students' scientific literacies, with a focus on students’ understanding of open scholarship more broadly. For example, Toelch & Ostwald [68] designed and evaluated a hands-on postgraduate course on integrating open and transparent research projects into students' local research projects. Over 60 h in the course, students were introduced to the ethos of open research as well as practices including pre-registration, FAIR (Findability, Accessibility, Interoperability and Reusability) data sharing, version control and open access outputs. Half of the students enrolled on the course responded to an evaluation six months later, which found that students were generally positive about open scholarship and the hands-on research project element of the course. Further, 80% of the students agreed that using open practices would improve the quality of their own research. In this example, Toelch & Ostwald [68] provide a useful example of how learning open practices may confer advantages to students’ scientific literacies when embedded across the curriculum.

There were useful case studies of how embedding a concern for *replication*, as a second example of a commitment to open scholarship, may also influence students' scientific literacies (e.g. see [80]). The Hagen Cumulative Science Project [49], for example, was initiated as a systematic mechanism for German undergraduate students to conduct replication studies as part of course requirements of completing a research thesis project. In this project, students pre-register their projects, share their data and analysis code and are encouraged to use open-source statistical software. While Jekel *et al*. [49] did not directly evaluate students’ learning outcomes and impact, the authors noted that at the time of writing, more than 80 replication thesis projects had been conducted, suggesting that students had successfully learned about and used open practices to pass their thesis work. This represents the value of hands-on training which promotes scientific literacies. Similarly, Collaborative Open-science REsearch (CORE; [81]) is a mass-replication-extension project in judgement and decision making and social psychology, with students from University of Hong Kong Department of Psychology and international early-career researchers. This project, which involves students conducting collaborative studies with hands-on support, has resulted in 20 peer-reviewed publications (e.g. see [82–84]). This project also has positive student evaluations [85]. Therefore, beyond the reported career benefits of publishing peer-reviewed journal articles, students also developed their scientific literacies, including analyses and coding with reproducible open-source softwares (e.g. Jamovi and R), analyses and assessment of original articles (e.g. detecting errors, effect size and confidence intervals calculations), power analyses (e.g. using G*Power or R packages) and rigorous interpretation of results (e.g. not over-relying on *p*-values) during the process. In this evaluation, scientific literacies were not directly measured quantitatively, but the product of this replication-extension project was generally well recognized by the scientific community for its high reproducibility, transparency and rigor; for example, the project has been included in summaries of good practice in large-scale replication efforts in the literature (see [86]).

Furthermore, integration of replication and reproducibility studies can be a useful way of developing students' scientific literacies; Smith *et al*. [64] integrated replication studies as part of academic training for 24 graduate students. In this initiative, students reproduced the results from instructor-selected high-impact papers in biostatistics using the original authors’ raw data and reported statistical methods. Based on their experience, in a mixed-methods survey evaluation, students reported enhanced confidence in data analysis, more exposure to new statistical methods and increased practice with scientific writing. In qualitative open-ended questions, students also reported an increase in their self-confidence with data analysis and provided positive comments regarding the overall experience. This is particularly encouraging, given the widespread prevalence of statistics anxiety among university students (e.g. [87]). The potential for this type of pedagogic approach to mitigate such anxieties and to build students' confidence in analysing quantitative data merits further research. This demonstrated the value in replication as a pedagogic tool that enhances students’ scientific literacies.

Beyond replication and reproducibility, there were other examples of embedding open and reproducible scholarship into teaching and learning, through innovative, active-learning approaches to help students understand aspects of the research process (e.g. [58]). For example, Sawchuk [63] reported an evaluation of an active-learning activity, whereby small groups of undergraduate students replicated a published study which investigated contents of age-specific birthday cards. Active learning was defined in this study as ‘instructional activities involving students in doing things and thinking about what they are doing’ ([88, p.iii]). Each group of students was asked to analyse a set of 15 different birthday cards and to share qualitative and quantitative findings with classmates before submitting a written discussion section on their results to the course instructor. This was a replication of a published study which used an identical analytical approach. While there was no formal evaluation of the project's impact, the authors note anecdotally that student engagement appeared to be high and that down-stream consequences also involved greater learning and meeting learning outcomes of the course. However, more formalized measures are needed to make a claim about the benefits of the exercise. A second example is Altunoglu [39], who also emphasized how using open-education learner management systems can improve student literacies, by making students ‘active participants in their own learning’ (p. 96), which also highlights how open tools may increase active learning in teaching and learning contexts.

Furthermore, our review identified examples of larger scale, collaborative approaches in supporting students' scientific literacies. For example, the CREP [89] supports students in conducting a replication project, under the guidance of a supervisor and with oversight of the CREP team. Students gain experience of tools such as the Open Science Framework, an internal peer review process, pre-registration and open data and code. Students contribute their data to a metaanalysis which can, potentially, result in authorship on published papers. Thus, students receive authentic training in relevant research skills within a context that is analogous to the wider research ecosystem. Wagge *et al*. [89] noted that more than 120 student groups had begun a project through CREP, demonstrating widespread interest in the value of replication-based research training. In general, authors also note that involving students in fully realized research projects, even from the first undergraduate year, may lead to improvements in general research skills, increased interest in a science-focused degree, and course engagement (as per [66]). This is aligned with the values of open and reproducible scholarship, because it promotes the principles of collaborative, transparent approaches to research (see [90], for another example). However, these predictions about the pedagogical potential of CREP require empirical evidence to substantiate them.

Open scholarship training can also benefit students’ skill development. For example, Steinhardt's [65] experience of teaching open practices for qualitative work suggested that the wider educational context may impact development of scientific literacies. In Steinhardt's [65] case study, students generally used practices and skills when they were mandatory but did not go above and beyond requirements. It may be that when the educational system encourages students to be producers of knowledge and of learning, development of research skills such as open and transparent practices can be most successful. A note of caution comes from Sacco & Brown [60] who found that an educational intervention to reduce acceptability of QRPs among postgraduate students (*n* = 49) was effective one week later, but that acceptance of QRPs had risen two months later (though not quite as high as pre-intervention levels). This was measured quantitatively by asking participants to rate 31 different QRPs on their defensibility. These authors note that repetition of such training might be needed to maintain scientific literacy. Thus, it may be that training in the use of open and transparent research practices should be repeated or maintained over the longer term.

### 3.2. Students’ consumption of science

In addition to students' scientific literacies in terms of how students *do* research themselves, *consumption* of science also forms an important part of scientific competencies. We conceptualize consumption of science as students’ ability to consume, critically appraise and evaluate research findings. Our review demonstrated that there is a plethora of peer-reviewed articles in this area. Haas & Rouse [91], for example, recommend that students be introduced to the concept of correction in the scientific record and taught how to identify and interpret correction notices during a literature search. One of the key requirements of a reliable scientific record is that publishers issue corrections whenever substantive errors in published research are identified. Complicating this picture is the variety of terms used to refer to corrections, including errata, corrigenda and retractions. Understanding the role of corrections provides necessary knowledge regarding the academic publishing process, while also introducing concepts such as fallibility and responsibility for ensuring the accuracy and completeness of the scientific record.

Related to this, there were accounts in our review whereby students were encouraged to understand the *process* of research itself. For example, Marshall & Underwood [53] describe a project that was a component of an upper-level undergraduate economics writing in-the-discipline course and that can be adapted in different undergraduate economics courses. The objective was for students to develop an understanding of how economists conduct applied empirical research. They used a number of tools and programs for statistical computing, word processing and providing feedback, and assignment submission and return, with reproducibility in mind. Each of these tools aimed to help students connect their discipline-specific writing skills with applied empirical research. While there was no formal evaluation, in their reflection of the project the authors note that the course can ‘promote student learning’ and leads to ‘organization and coherence’ throughout the writing process. Further, drawing on their experience from methods courses taught to undergraduate and graduate students, Frank & Saxe [92] argue that students should replicate recent findings as part of their training in experimental methods. In their own courses, the authors found that replicating cutting-edge results is exciting and fun for students; it gives them the opportunity to make real contributions to science and provides understanding of the scientific process, the importance of study reporting standards, and the value of transparency and openness. One of the benefits of doing replications to students is that they learn much from carefully reading others' papers ‘*with the eye of a replicator, not just a reader*’. The authors recommended that reading scientific papers with the goal to replicate helps students appreciate transparent and complete scientific reports, although these claims now require empirical scrutiny.

### 3.3. Development of transferable skills

There was also evidence to suggest that open and reproducible scholarship can impact development of certain transferable skills for students. The advancement of students' scientific literacy directly promotes the development of closely related skills transferable into contexts outside of academia. The ability to work with quantitative data, draw accurate conclusions and understand the limits of inferential methods is a widely sought-after skill-set in the industry [45]. By engaging in open scholarship, students can develop the ability to work as part of a team—whether in the area of collaborative coding, problem-solving or co-production of knowledge [44,45,52,66]—while also fostering a sense of independence and agency of their own learning process [41,59]. Furthermore, students who receive training in reproducible scholarship may also be better equipped with public science communication, because they have received training in communicating research findings in a transparent and accessible manner [41,93].

Our review also highlighted that students can benefit from the teaching skills and the understanding of how education in general is structured [40,59]. For example, this can be relevant for those directing themselves to education-related careers or can be transferred to any training or coaching context in other career fields. Andone *et al*. [40] in particular charge a small number of students with creating open educational resources as a way to enrich STEM undergraduate and postgraduate programmes and, at the same time, as an assessment from which students gain knowledge on best practices in education content creation. The qualitative data gathered from Andone *et al*.'s participating students (*n* = approx. 120) demonstrated that 94% of them found the practice useful and applicable to a number of course elements (e.g. for theoretical knowledge transmission, independent knowledge evaluation and study).

Scientific literacy skills can be valuable in careers where the communication of scientific findings to stakeholders with non-science backgrounds is imperative. For example, Baran & AlZoubi [41] conducted semi-structured interviews that explored students' perspectives of open pedagogy (*n* = 13). These interviews demonstrated that students found learning about open scholarship benefited their ability to critically analyse science as well as improving their understanding of broader ethical issues such as trust and integrity. One of the most important potential benefits of open scholarship education is the development of reproducible coding and data analytical skills, which may be helpful for future careers.

Similarly, Çetinkaya-Rundel & Ellison [45] presented an account of the benefits of a data science course (which is different from typical statistics courses, as it emphasizes open source/data and reproducibility) and argued that the course can help students in developing reproducibility routines, specifically version control and coding collaboration skills with Git and Github (through working with other students on assignments), which are transferable skill-sets desired by future employers. Çetinkaya-Rundel & Ellison [45] also mentioned that the course satisfies statistics requirements for students in a wide range of different disciplines and students were allowed to work on any dataset for their final project.

In addition, Andone *et al*. [40] find that students’ participation in open educational resource creation enriches their digital lifelong learning abilities. Their study (*n* = 120 undergraduates) showed that a high majority of the participating students claimed they had to learn new technologies in order to accomplish the task of open educational resource creation, and the tools used varied, due to the creative nature of the task.

However, there is also evidence demonstrating *little* impact on student outcomes, in formal evaluations of students' future research behaviour. For example, Nurse & Staiger [94] evaluated the impact of a service-learning activity where students analyse real-world data on behalf of non-profit organizations. An evaluation comparing students who did the activity (*n* = 22) versus a comparison *(n* = 18) showed that this exercise failed to find evidence for an impact on students’ attitudes, confidence or planned research behaviours over and above the effect of simply attending lectures about reproducibility. These outcomes were assessed based on the degree of participant agreement with a series of statements (e.g. ‘we should trust social scientists to be honest about how they analyse their data’.) measured on a Likert-type scale. However, qualitative comments indicated that students who participated in the service-learning activity were better able to describe the different elements a journal might require for reproducibility (e.g. command files and a data appendix) in comparison to a control group, suggesting an increase in scientific literacy relating to reproducible data analysis.

Finally, our review demonstrated the value in positioning students as co-creators, or co-producers, in a research context, which aims to strengthen students' ‘hands-on’ research training and encourage them to be active researchers in their curriculum. For example, Ryan [59] developed a hands-on research training programme and evaluated it using module evaluations and opportunities for students to demonstrate learning outcomes (either via an oral interview or written assessment). Qualitative comments in module evaluations and module assessment transcripts demonstrated that the programme improved students' sense of ownership and independent thinking with their research practices (see also [44]). This theme was also discussed by Baran & AlZoubi [41], who developed a qualitative analysis of student reflections following an open education resource-based module (*n* = 13 undergraduates). The authors demonstrated that students qualitatively felt a strong sense of agency and ownership of their own learning and knowledge [41]. Similarly, as discussed by Button *et al*. [44], restructuring typical undergraduate research projects to be larger, better powered, collaborative efforts of a consortium of students not only taught students about the principles of high-quality transparent research but also allowed students to benefit from joining a ‘knowledge creating community’ [44, p. 85]. A similar approach was described by Stiemsma *et al*. [66], who developed a ‘Students as Scholars Program’. In turn, this encouraged the development of highly sought-after transferable skills such as teamwork, problem-solving and co-production of knowledge (see also [44]). The pedagogical outcomes of this approach, in more empirical terms, now warrant investigation.

### 3.4. Interim summary: students' scientific literacies

From this prior research, we can conclude that many pedagogical approaches aim to increase literacy, which is viewed as an important goal for students' learning. There is some evidence to suggest a positive impact of open and reproducible scholarship but other evidence also suggests that impacts are weak or short-term; thus, further evidence on actual measurable outcomes (and, indeed, which methods and topics produce the most impact) is now needed. Further, the majority of the research considered approaches to teaching and learning open and reproducible scholarship that aimed to bolster students’ understanding and competencies, including research competencies and statistics literacies. Importantly, and perhaps most unexpectedly, our review demonstrates that there is some emerging evidence that embedding open and reproducible scholarship can impact non-discipline-specific skills, including transferable skills such as writing, teamworking and problem-solving. Therefore, this broadly suggests that, while there were some studies which found little effect of integrating an open approach, open scholarship in teaching and learning contexts can be useful for students' skill development and that this goal is recognized as important by those in the field. This includes both research-specific skills, such as statistical understanding and knowledge about open scholarship, as well as more transferable skills that may be useful in employability contexts.

## 4. Open and reproducible scholarship and student engagement

The second thematic category that our review covered is 'student engagement'. Indeed, the process of developing students' research competencies and improving their scientific literacies would be impossible without a good level of student *engagement* with teaching and learning about research. Student engagement is thus an important, yet often-overlooked, facet of the student experience in the context of research training, perhpas owing to an inherent focus on academic achievement as the sole measure of ‘student success' across the sector (e.g. [95]). Therefore, the student engagement literature is smaller and less developed than the research literature which investigates competencies and literacies. Further, student engagement is also a complex term that differs between subdisciplines, but it broadly refers to ways in which students are involved with and enjoy their academic studies. Thus, the term is typically related to more subjective outcomes (e.g. student satisfaction and wellbeing) as well as some objective measures (e.g. retention and academic success; see [95]). Our review highlighted many examples of how teaching open and reproducible scholarship seeks to encourage student engagement, including a focus on (i) students' motivation and engagement with their learning, (ii) the value of collaborative, student-centred approaches to open scholarship teaching and (iii) the impact on students’ engagement in future open research behaviours. These three facets of engagement are largely aligned with Groccia's [96] model of student engagement, which notes how there are three distinct areas of engagement: affective (e.g. interest, enjoyment and motivation), cognitive (e.g. focus, concentration and reflection) and behavioural (e.g. effort, persistence and dedication) engagement. We now synthesize the evidence across these perspectives.

### 4.1. Motivation and engagement with learning

Our review highlighted that student motivation and engagement may be achieved through the integration of open scholarship tools into teaching. For example, Frank & Saxe [92] make a compelling case for the notion that replication of studies with students can be an opportunity for exciting ‘hands-on’ research training, which gives students the chance to contribute to scientific research and provides lessons about the scientific process. The PsyTeachR project at the University of Glasgow (see [97]) has embedded open software R and RStudio to help students learn reproducible and robust methods with real datasets; in an evaluation, students found the experience of learning R exciting and helpful [98]. Similarly, Button *et al*. [44] reported in a descriptive commentary drawing upon student feedback forms that students ‘greatly valued participating in a consortium-based model for training students in conducting reproducible research projects, particularly having access to a meaningfully large data-set; the opportunity to network with academics, students, and researchers from other universities; the sharing of ideas and knowledge; and contributing to pre-registration’ [44, p. 86]. This further evidences the engagement value of adopting such an approach with students.

Another example of tools and practices aligned with open and reproducible scholarship is Davis and Parmenter's [47] embedding of a participatory action research (PAR) approach within pedagogy. This approach centres around working collaboratively with students to co-create research and understanding. Davis & Parmenter [47] used questionnaire, diary and semi-structured interviews, with a small group of students (*n* = 7) and staff (*n* = 2) and reported that the PAR approach makes students feel safe and accepted in a community, where they can freely express themselves. This was captured using a range of triangulated methods, including reflexive questionnaires, interview diaries and research diaries. In addition, participants qualitatively reported changing their perceptions of education and their expectations and goals for their own educational path (i.e. considering further education, gaining interest in more subjects and perspectives, feeling more credible and capable of contributing to the development of knowledge). This confidence and feelings that the students can contribute to knowledge both in education and in their future professional fields can build in learners an approach of inquiring and questioning knowledge more effectively. Similarly, students' affective engagement is also promoted through use of open educational resources. For example, Afolabi [37] used a pre-post-test design to show how such resources are seen as an advantage by students, who strongly agreed with statements such as ‘[the resources] make learning more meaningful’ and they are ‘a positive innovation’ [37, p. 117]. Werth & Williams [71] also qualitatively investigated the effect of pedagogy informed by open educational resources on students' motivation and engagement with students’ learning (*n* = 12 students). The involvement of students in co-creating the educational resource that would be available to their peers in future years was shown to increase student motivation and confidence with the content, and this was captured using semi-structured interviews with students. Likewise, Paviotti *et al*. [56] reported high student enjoyment and satisfaction (*n* = 24) regarding the design of a tourism course delivered through the use of open education principles.

A further example highlighted by our review is Lin [51], who investigated student perceptions of open educational materials compared to traditional textbooks within an introductory education course (*n* = 46 undergraduates). Survey and focus group data revealed that the majority of students (survey respondents 84.7%; focus group members 86.2%) viewed the open materials favourably, highlighting (i) dynamic and plentiful materials beyond what would be typically contained in a textbook, (ii) the ability to access the materials anywhere digitally and (iii) the cost-saving factor for their education. Sanchez *et al*. [61] also investigated students' (*n* = 144) perception of open educational resources, over the course of one year, on a criminal justice course. Qualitative student feedback showed two emerging topics: (i) feelings of relief and (ii) perception of accessibility. Students reported feeling relief mainly due to the high financial costs and limited availability of resources in North American institutions. In addition, the results reported that the majority of students (80%) reported perceptions of increased accessibility of the resources, allowing them to study, practice, annotate and transport resources more efficiently. Accessibility was also related to allowing visually impaired students to interact with the digital resources more efficiently than with traditional printed books. Similarly, Watson *et al*. [70] examined use of a free, open access online textbook and showed that students (*n* = 1299) highly valued the quality, features and cost of the online textbook. Further, by integrating open educational resources in teaching and learning, this resulted in clearly articulated learning outcomes, a fully realized structure in the course learning management system, and improvements in classroom practice. Students thus clearly benefit from the use of open educational materials.

It is important to shed some light into how open and reproducible scholarship may help overcome certain barriers to students’ engagement, such as those experienced by marginalized or under-represented students. For example, Bangera & Brownell [99] describe a ‘course-based undergraduate research experience’ (CURE) initiative, which aims to provide under-represented students, women, and those of low socio-economic status, with ‘hands-on’ science training. The authors describe how students who are under-represented and are first-generation university students face barriers including awareness of cultural scientific norms, access to opportunities, and perceived barriers to interactions with faculty members. The CURE initiative uses course-based undergraduate research experience to prompt engagement with science among under-represented students and, by extension, allows the exploration of ‘questions with unknown answers to expose students to the process of scientific discovery’ [99, p. 604]. Thus, open scholarship practices can have an important role to play in promoting wider engagement with science among students.

Further, knowledge of the practicalities of conducting open scholarship can also positively impact students' attitudes towards gaining knowledge on important issues, such as copyright law and integrity. For example, Hare *et al*. [48] created a curriculum centred around the production of an open educational resource for postgraduate students in the field of education (see also [38], for a similar intervention). Beyond teaching about open access pedagogy, the curriculum aimed to improve knowledge surrounding topics such as information privilege, intellectual property, access and copyright. However, there should now be empirical evidence to corroborate the effectiveness of this approach.

### 4.2. Collaboration and student involvement

Moreover, our review demonstrated how students' attention and reflection can be enhanced by open scholarship via adopting more collaborative approaches to student research. For example, Button *et al*. [44] found that embedding a *team science* approach to teaching meant that students anecdotally felt less isolated and more valued as part of a team. Clark *et al.* [100] also describe how students benefit from working with peers; the authors created a Peer Research Consultant programme, which trained students in research support, and found that students enjoyed seeking research assistance from peers over librarians. As Button *et al*. [44] argue, collaborative, team-based approaches to research with students could improve students' comfort and creativity with research processes (see also [31,101]), but more empirical research is necessary to corroborate this notion. More detail on the team science approach which elicited these positive attitudes towards participating in team science, and the knowledge exchange that results from it is described in Button *et al*. [44]. Similarly, in another example, Poronnik & Moni [57] used the team science approach to improve undergraduate students' science communication skills via the task of collaboratively writing opinion editorials, followed by an open and transparent peer review process. Poronnik & Moni [57] evaluated this approach using survey data with Likert-style quantitative questions (*n* = 230 students) and found that students generally appreciated the experience and found it cognitively challenging (80%) yet valuable (70%); so, overall, the team approach was considered as a rewarding learning experience for students and enhanced cognitive engagement, wider collaboration and more hands-on student involvement.

Both Button *et al*. [102] and Pronnik & Moni [57] describe approaches which embed open and reproducible scholarship through working in partnership, or engaging in co-creation, with students. This appears to be a particularly useful mechanism to improve engagement. Such a procedure is described explicitly by Ryan [59], who developed the ‘*students as researchers of their curriculum’* (SAROC) approach. Ryan [59] then used the SAROC adoption to investigate the reflective thinking and understanding of students in a teacher education programme, calling into question what students perceived *being a researcher* to mean, along with other learning outcomes. The impact of this approach was qualitatively investigated; through a thematic analysis, a small group of students (*n* = 8) were recruited to evaluate the SAROC programme. This evaluation demonstrated that students' use of self-reflection and understanding of the research process was improved via the SAROC approach. When students worked as researchers and co-creators of their curriculum, this helped them to reflect critically upon the whole research process, be more engaged in the research, and generally start a process of ‘*becoming researchers*’ [59, p. 644] also suggested that the SAROC can lead to the development of ownership and independent thinking in education, which are explicitly related to the cognitive aspects of student engagement.

### 4.3. Engagement in open research behaviours

Implementation of open and reproducible scholarship tools may also positively influence the down-stream behavioural aspects of student engagement, such as enrolment in the course, engagement with the learning materials, and future uptake of open research behaviours. For example, Çetinkaya-Rundel & Ellison [45] observed that introducing open educational resources within a data science course led to increased enrollment in the class in subsequent years. Further, Sanchez *et al*. [61] adopted open educational resources and evaluated this with students using qualitative methods. Students in the evaluation (*n* = 144 qualitative comments) noted that the use of open resources was also more accessible and easier to engage with, which improved students' overall engagement with the materials. Therefore, this suggests that integrating open materials, as well as an explicit concern for open research *itself*, can also be a useful way of improving student engagement in a teaching and learning context (see also [103]). As well as accessibility, combining open educational resources with further open pedagogy in teaching has also been found to increase student engagement through increased perception of agency. Tillinghast *et al*. [67] investigated perceived differences between using only open resources versus combining open resources with an open pedagogy in teaching, including open scholarship specifically (*n* = 127 students; *n* = 113 in a survey; *n* = 9 in interviews). Overall, they found that, using a sequential mixed-method design, there was not much of a numerical difference in engagement between the two, as both were perceived as being of good quality and easy to use. However, expressions of increased student engagement were noted across qualitative interviews.

There was a plethora of research in our review that discussed the consequences of using open educational resources specifically. For example, Bloom [43] randomly assigned students in an introductory English course to one of two forms of assignments; students either edited openly accessible resources to create learning tools for others’ benefit (*n* = 60) or completed traditional assignments (e.g. an essay) in which the created materials have no value other than being graded (*n* = 32). Students editing open educational resources on average produced somewhat fewer (4.9 versus 5.8) examples in an essay, although a formal result of a statistical test was not provided, and there was no evidence for differing performances between the groups in an end-of-module quiz. Potential reasons were not explored; however, due to divergences in the course content because of the different forms of assignments there were also differences in the requirements for the essay, which might have impacted the results. Nonetheless, this is tentative evidence that working within an open educational framework does not negatively impact students' learning compared with the conventional approach in which students are far more experienced.

Finally, beyond the undergraduate experience, there was also evidence here to suggest that open scholarship may impact postgraduate students, such as doctoral students. For example, Hare *et al*. [48] also reported increased engagement in 34 doctoral students in education learning about open access; for example, the authors found developing mastery to be the dominant theme in qualitative evaluation, which they describe as reflecting students’ engagement with the content in a process of sense making. Although a small portion of the text data conversely suggested that students demonstrated *resistance* to take steps towards open scholarship, the authors' interpretation suggests that this might be underlined more by a lack of self-confidence, as opposed to a lack of engagement.

### 4.4. Interim summary: student engagement

Overall, our review demonstrates that use of open educational resources and the collegial work environment can facilitate students' personal growth as *science communicators* and *research collaborators* [104] and open scholarship practices can empower (with skills and competencies) and inspire students (with motivation) to engage as *science contributors* who conribute to the scientific knowledge [42]. While the popularity of studying the impact of open scholarship practices and especially using open educational resources in undergraduate teaching has increased in recent years, research in this area is sparse, lacking systematic approaches as to how open scholarship affects: (i) the different aspects of student engagement including the behavioural, affective and especially the cognitive aspects of student engagement (e.g. focus, attention and concentration); (ii) the ways students can engage academically in learning, teaching and research; as well as (iii) students' surroundings for active engagement including peers, faculty and community [96]. Furthermore, research on teaching open scholarship does not yet reflect the broad spectrum of open scholarship practices and, to date, has mainly focused on teaching reproducible computing, e.g. using open-source software and version control. In this section, we have summarized the existing evidence on the link between the open scholarship and student engagement in research and mapped the areas of further investigation.

## 5. Open and reproducible scholarship and students' attitudes towards science

Finally, the third thematic category identified by the literature review and driven by our research question was the outcomes associated with open and reproducible scholarship on students' self-reported *attitudes towards science.* We broadly defined attitudes as individuals' positive, negative or neutral feelings about certain behaviours, topics or practices [105]. As open scholarship norms and practices have emerged and changed dramatically over the last decade [1,2], the attitudes of academics and researchers towards initiatives and reform surrounding transparency and rigour have been diversifying, evidencing hope for transformative change, and concern surrounding the practicalities of implementation (e.g. [106,107]). In this section, we present and synthesize the empirical findings on students' attitudes towards practices of open and reproducible scholarship, reflected by their self-reported feelings towards, and perceptions of, engaging with such practices.

### 5.1. Students’ attitudes towards science

Our review demonstrated that although students' attitudes towards science can be shifted through one stand-alone class, these changes are not necessarily large or temporally stable, nor do they always translate to behaviour change. For example, Chopik *et al*. [46] reported a significant modest decrease in psychology students' trust of the scientific work done by psychologists following a 1 h lecture on the replication crisis (*n* = 194 students). This was measured using a pre-post survey. Furthermore, this work evidenced no impact on the students' intentions to pursue graduate study, suggesting that student recruitment and career pathways are unlikely to change. Similarly, Sacco & Brown [60] investigated the effect of a 1 h training module on QRPs, targeted at psychology graduate students. The module covered the implications that QRPs can have across science, the public and researchers' reputation. One week after this module, students reported trusting the findings of psychological studies significantly less. Importantly, Sacco & Brown [60] reported that the effects of training graduate students to identify and evaluate QRPs are transient, with benefits diminishing in the two months after the training session. Therefore, more sustained efforts embedded within programmes may be required in order to maintain students' awareness/concern in the longer term.

Furthermore, there is evidence to suggest that embedding open scholarship tools into teaching and learning contexts does not always translate to subsequent behaviour, in the context of attitudes towards science. For example, Marwick *et al*. [52] integrated replication across an empirical archaeology course. After a class assignment that centred around replication, students (*n* = 13) reported in a module evaluation that while they perceived the value of replication for archaeology in general, they do not see any specific benefits to doing it themselves. While students often understand the wider implications and importance of replication work, the implications for themselves, and their own research skills and careers, are infrequently discussed.

There was also further evidence in our review of the effects of embedding open and reproducible science across full modules, syllabi and assessments. For example, Hanna *et al*. [108] designed an undergraduate module that introduced students to open research and reported that students (*n* = 72) expressed a general positive attitude towards open practices after taking the module, in a free-text module evaluation using qualitative comments. Hanna *et al*. [108] also identified that key benefits, in terms of student attitudes, were for transparency, collaboration and research progress. Truan & Dressel [109] also investigated attitudes towards and experiences of students with practices of open education, through a seminar about research-based linguistics. Students (*n* = 59) produced written narratives and then completed a quantitative survey on (i) their willingness to upload and publish academic-based posters in an open access format, (ii) teaching concepts in the form of open educational resources and (iii) their own reflections on their personal experience of engaging with open access publishing. Qualitative analysis focused on students' motives to publish their work using open practices. Results evidenced positive attitudes towards open scholarship practices and students' key motives to use such practices related to a sense of belongingness, personal educational rewards and active engagement in a collaborative spirit. However, students also reported attitudes related to their fear of visibility and copyrights of their work, as well as concerns about licensing.

### 5.2. Assessment of scientific quality

Beyond reported trust in, or attitudes towards, science, open and reproducible scholarship in pedagogy may also impact how well students are equipped to make assessments of scientific quality and credibility. Again, our review demonstrated that the implementation of open and reproducible scholarship can have benefits to postgraduate, as well as undergraduate, research training. For example, prompted by the current crisis of low confidence towards psychological science, Sarafoglou *et al*. [62] introduced a Masters level course for research students, which discussed good research practices and was structured using the book ‘*The Seven Deadly Sins of Psychology*’ [26]. The course included topics related to procedures of replication of studies, steps for pre-registering current research and open public sharing of data and analytic procedures, and students were given the opportunity to engage in active discussions about open scholarship practices.

Student evaluation in a module feedback survey (*n* = 43 Masters students) demonstrated that students' attitudes and feedback were positive and that students showed interest in discussions on these themes, as well as appreciation of the importance of teaching practical skills of open scholarship engagement. On the other hand, students also reported that they felt pessimistic after learning about the replication crisis and a crisis of confidence towards psychological science. This indicates that information about low replicability may reflect a more accurate perception of their field, however, it may have unintended consequences for students’ attitudes towards science. Finally, students reported an increased sense of responsibility in dealing with research projects, where they have the opportunity to apply open scholarship practices. There is clearly a need for targeted efforts to improve graduate students' assessment of scientific quality. For example, Pan [55] explored graduate students (mis)perceptions about responsible conduct of research. Graduate students in this study (*n* = 518 across Taiwan and the USA) were given a test which measures students’ justification of ethics and research responsibility. Findings suggested that graduate students were generally able to judge the ethical acceptability of scenarios in the test but could not always explain their judgements; while conclusive inferences cannot be drawn from one sample, this may indicate a gap in current training provision in some contexts.

One important aspect of students' research training is through their undergraduate thesis, which represents a promising opportunity for students to change their understanding and attitudes to science. Krishna & Peter [50] investigated the attitudes of German students towards QRPs and use of such practices in their Bachelors and Masters Theses research (*n* = 207). Overall, perceptions of supervisors’ attitudes towards QRPs predicted variance in both attitudes towards science and use of QRPs. Interestingly, while supervisors' perceived attitude was a significant predictor, neither supervisors’ perceived expectation of the positive results nor the students' belief that positive results constitute superior science were related to the students’ engagement in QRPs. On a more optimistic note, the authors concluded that while the self-reported prevalence of QRPs among student researchers is somewhat comparable to those more senior, their endorsement of these practices is lower. As they sum up: ‘*early-career psychological scientists do not like QRPs, but may still feel pressured to use them*’ ([50, p. 17]). Considering the attitudes of early-career researchers towards QRPs, and the direct relationship between their endorsement and engagement in QRPs with perceived supervisors' attitudes, it is reasonable to assume that direct mentoring could play a pivotal role in shaping current and future research practices of undergraduate and graduate students. This work clearly highlights how Higher Education structures and power dynamics, such as those between staff and students, can either facilitate or restrict the uptake of open research behaviours. A similar conclusion is reached by Olsen *et al*. [110], who argued for the promotion of open scholarship in economic postgraduate student supervision, in order to foster critical reflection on scientific literature. However, despite these recommendations (see also [79]), our review also showed that there is little empirical work that directly investigates how incorporation of such an approach can impact student outcomes.

### 5.3. Interim summary: students’ attitudes towards science

To summarize, our review tentatively suggests that efforts to increase awareness and understanding of open scholarship are capable of modestly influencing students' attitudes towards science. Furthermore, applied tasks appear to have additional value for supporting practical experience and skill development beyond lectures or information-giving interventions. However, much of the work presented here relates to modest pre-post-intervention comparisons with insufficiently validated attitudinal measures, limited use of control groups and longitudinal designs to determine sustained effects, and evaluates practices which are highly contextualized in local environments. Given such deficits across the literature, collaborative attempts to conduct robust evaluations should be prioritized to provide robust and convincing evidence for impacts of open scholarship upon student attitudes towards science.

## 6. Discussion

This review of the literature aimed to critically synthesize the existing evidence on how embedding open and reproducible scholarship in teaching and learning may impact student outcomes, including students’ scientific literacies, student engagement and attitudes towards science. Our review has demonstrated the current literature in this area and generally suggested that embedding an open and reproducible scholarship approach into teaching may confer advantages to students in the short, medium or long term. We found evidence to suggest that adopting an open and reproducible scholarship approach can positively impact the student experience, most notably in areas such as strengthening students' scientific literacies and offering creative ways for them to engage with their research training. However, we also highlighted that the strength of evidence can be improved throughout, and there are examples in our review where adopting an open and reproducible approach did not impact students’ behaviours. This review ultimately aimed to contribute to the appreciation of how open scholarship may have impacts in teaching and learning contexts, which is a necessary pursuit to continue advancing the robustness, reproducibility, openness and transparency of research and scholarship.

However, crucially, our review also highlighted that the majority of literature in this area lacks methodological and analytical robustness^2^. Indeed, for us to make comprehensive and more causal conclusions from this literature, the values of rigorous, thorough, transparent and methodologically robust science should be applied to the pedagogical evidence base. For instance, some empirical studies identified by our review had no control groups, and this lack of experimental manipulation means that it is difficult to draw causal conclusions as to whether responses from students were a *direct* result of inclusion of open scholarship practices. Similarly, there were very few interventional or experimental studies; the majority of this literature centred around narrative accounts of teaching practice, with a lack of control groups and intentional study design. However, it is also worth noting that experimental studies may be logistically challenging in pedagogical contexts. For example, there are practical and ethical considerations that make establishing a ‘true’ control group of students who do not receive teaching interventions difficult. Further, there are inherent confounds of lecturer or instructor characteristics, in that instructors who are passionate and interested in open scholarship may engender a more engaging student experience, irrespective of the teaching content. In addition, our review identified a general lack of reporting standards across this literature. For instance, some of the studies in our review did not report any numerical results, instead focusing on anecdotal feedback, and some sample sizes were unjustified or small. This may be problematic for reproducibility, making findings less verifiable, credible and informative. To provide context and wider implications to each section of this review, we now provide summaries of the core findings across the three themes of this review, before discussing limitations of this evidence and implications for practice.

With the cultural landscape of an increased emphasis on *scientific literacies* as a core output from Higher Education, our review generally concluded embedding open and reproducible scholarship into teaching can impact students' ability to *consume* and *conduct* scientific research. Consumption of scientific research involves reading, critically appraising and evaluating research findings. Conducting research includes competencies in actions, such as research design (e.g. experiment preparation or data collection) and data analysis (e.g. statistics). Using this as a broad framework, our review found three broad areas under the scientific literacies umbrella category that can be impacted by the implementation of open and reproducible scholarship: (i) *the understanding of open research, (ii) the consumption of science* and *(iii) the development of transferable skills.*

Several reports on the implementation of open scholarship pedagogy (e.g. CORE and CREP) demonstrated that introducing open and reproducible methods can increase understanding and consequences of responsible research practices, as well as support undergraduates to *conduct* open research. Thus, there is evidence that embedding open and reproducible scholarship can help students’ consumption of science, through increased understanding of the research process, peer review, and statistical analyses used in the published research. Open and reproducible scholarship can also aid the development of transferable skills by giving students opportunities to work with real ‘messy’ data, draw their own conclusions from real data and thus begin to understand the strengths and limits of different statistical methods. Additionally, open scholarship can create new opportunities for students to develop other skills, including teamwork, communication skills, scientific writing and to foster a greater sense of agency over their research. Such skills are useful for students in many careers, but invaluable for those entering academia [111].

Second, our review demonstrated the potential value of embedding open and reproducible scholarship into teaching for *student engagement,* including factors such as motivation, confidence, collaboration and co-creation. In line with these factors, a key theme repeated throughout the literature was the notion that open scholarship can improve student engagement through lowering barriers to participation across Higher Education, particularly with research. Notably, the widespread use of open-source learning materials can make valuable knowledge more accessible to all. Furthermore, student involvement was greatly motivated due to the value that open scholarship teaching practices placed on opportunities for students to share ideas, build professional and personal networks, or upskill their research practices. This benefit was particularly prominent in under-represented communities. Open scholarship also widened the adoption of open research behaviours, which was most effectively achieved in consortium-based projects, where students work collectively with other students and staff across several universities on a project or large dataset using robust and open research practices. Similarly, our review also highlighted how research on the effects of open research practices on student engagement was mostly, although not solely, focused on the introduction of open educational resources. In many cases, use of student-curated open teaching resources increased accessibility of materials, perhaps especially for those of lower socio-economic status or with visual disabilities for whom access to regular materials was limited due to cost or inflexible formatting. Use of open educational resources can also involve the co-creation of course materials with students and faculty, which has shown increased student engagement in terms of collaborative activities and involvement with science.

Finally, we reviewed the literature that focuses on students' *attitudes towards science.* The reviewed papers found an imbalance between acknowledging the importance of open practices and implications for students’ behaviour. We found evidence that although single-teaching sessions might change student attitudes short term, much more considerable focus on open research practices is necessary to make sustainable change. While there is initial evidence that teaching open practices can enhance students' attitudes towards science, the students do not always think that open practices are relevant for their own work. Apparently, students have a similar attitude to more established researchers, reporting similar concerns (e.g. with copyright or licensing issues). Furthermore, two key components arising are the trust students have in science and their ability to assess scientific quality. These two aspects are not only important considering the future role of students as potential researchers or decision makers, but also as citizens. Making students aware of the replication crisis is certainly an important task. However, it is important to ensure that students do not complete courses with a decreased trust in scientific work, but instead, develop a positive attitude towards open practices as a means to avoid unreproducible research. Consequently, it is crucial to avoid a change from being critical about scientific findings (an important task in science) to losing trust in science.

### 6.1. Limitations

Our review served to provide a critical summary on the existing evidence and identify important works that explore how embedding open and reproducible scholarship into teaching can impact the student experience across academic disciplines. However, as mentioned above, our review highlighted that there could be improvements in the methodology, reporting and transparency of empirical research within the pedagogical literature that examines the impact of open and reproducible scholarship. Therefore, we recommend that scholars wishing to formally evaluate their pedagogy should employ more Team Science approaches, that focus on collaboration and data sharing, to offer a practical solution to the methodological limitations observed in the empirical studies. As a wider point, the robustness of the open scholarship evidence itself should be subject to wider scrutiny in future contributions in this area. Put simply, open scholarship research should adhere to the rigorous standards of open scholarship itself. We thus also recommend that future open scholarship research abide by rigorous standards regarding methodology, reporting and transparency, by, for example: (i) implementing control groups when possible; (ii) adhering to rigorous reporting standards and reporting clear and transparent results when possible and (iii) employing larger sample sizes and justifying sample sizes, when possible.

Second, in terms of limitations, it is important to note that our literature search showed that very few published studies report null or inconclusive findings; therefore, it should be noted that publication bias may likely influence the validity of the published literature. Publication bias is defined when the evaluation of a study's publishability disproportionately hinges on the outcome of the study with the inclination that novel and significant results are worth publishing more than replications and null results [112]. This is an issue because there is an inflated and disproportionate rate of positive significant findings in the traditionally published literature [113,114]. While the prevalence of publication bias across the social sciences is well established [12], this may extend to the literature on student engagement, open educational resources and open scholarship behaviours (e.g. data sharing and pre-registration). The claims of open scholarship and student engagement can be corroborated themselves by implementing open scholarship behaviours, such as pre-registration and Registered Reports (see, for example, [25]). These behaviours can act as a useful tool to quality check, verify and paint a more realistic picture about student engagement and open educational resources. For open scholarship to facilitate research transparency fully, all aspects relating to the scientific process (e.g. publishing data, materials and details needed for data analysis) are required to be made openly available [113]. As a result, and where possible, we encourage this field to adopt open scholarship practices to overcome publication bias and aid transparency.

Finally, it is important to note here that some of the literature highlighted by our review may be discipline- or context-specific, and not designed to be widely shared or applied in our contexts. For example, there may well be approaches shared here that cannot be transferred to other teaching and learning contexts, due to course accreditation requirements, staffing levels, and nature of the student cohort. Therefore, we encourage educators to take the findings of this review as a useful starting point, before critically and creatively considering how these findings may be applied within their own local contexts. Furthermore, we also appreciate that there is likely to be valuable and insightful evidence of the impacts of embedding open and reproducible scholarship that is not reflected in the formal published evidence identifiable by our review. While we made attempts here to locate grey and unpublished literature, there is likely to be evidence that supports (or, indeed, refutes) the claims made in this review that we are unable to access, for example, student evaluations or student surveys without consent to share. Therefore, we also recommend that educators and scholars consider interventions to promote and incentivize the sharing of student evaluations in ways that are ethical, thoughtful and robust. This may include, for example, wider emphasis on pedagogical research and evaluation in teaching and learning contexts.

### 6.2. Implications and recommendations

We hope that this review (i) provides educators with an evidence-based rationale for embedding open and reproducible scholarship within teaching practices, and (ii) demonstrates multiple ways that this goal can be realized across manifold teaching and learning contexts. Higher Education is fundamentally about creating and evaluating knowledge; educators create curriculums and courses, students develop understanding of this knowledge, and Higher Education communities are created. Team Science is an approach that can be used to develop this community, by allowing the student and staff network to interact more through co-production, or with peers and researchers at other institutions; to expose students to better practices; to feel less isolated; and to be valued as part of a team. As a result of this interaction, students are able to think deeper and be more up to date with research practices. Put simply, a positive attitude can be encouraged towards participating in Team Science and knowledge exchange. It is this development and transformation of knowledge that higher education crucially offers to society. Embedding of such an approach is possible with the use of open educational resources, which encourage accessibility of the class materials, allowing them to be easier to engage with. Open educational resources would allow us to transform how knowledge is integrated in teaching and learning and how it can be disseminated from professors to students, teachers and society. Finally, this would encourage the students to feel that they can contribute to their own learning and that they have agency and choice to learn as much as possible. Therefore, open educational resources can improve attitudes towards science and positive feelings about the project having a use beyond their module.

The findings of our review fit well with the Universal Design for Learning (UDL), a framework to improve and optimize teaching and learning for all people, based on scientific insights into how humans learn in Higher Education [115–117]. The evidence highlighted that choice should be provided to enable students to develop agency in their own learning. However, there are structural barriers that are encountered in this endeavour, particularly in the context of creating new open educational resources. Thus, open educational resources would be required to make learning more accessible and meaningful as opposed to being more work than help. For instance, elsewhere in the literature, lecturers have discussed the benefits of lecture capture [1,2,118], because it allows students to learn in an environment that suits them, to learn at their own pace and to develop their own speed [119]. Open educational resources allow students to have an opportunity to develop agency in their learning, thus be more engaged with the materials and be motivated in a similar way that scientists are being motivated to work on a specific problem. Open educational resources would allow the academic researcher and student to engage in a way that fits the teacher's style and the learner's preference that can improve creativity and new perspectives which would otherwise be ignored or not considered. Last but not least, open educational resources promote and facilitate social justice and equality. Thus, we reassert the importance of open scholarship, specifically open educational resources, as being a tool to improve our knowledge when thinking about student engagement, a form of UDL and the development of teaching and learning in Higher Education more generally.

It is worth noting here that we have focused our review on undergraduate and postgraduate students within Higher Education, on the basis that there is little-to-no evidence considering the potential implications for introducing open scholarship at other education levels. Outcomes are unlikely to be unique to Higher Education students; therefore, there may be some value of such pedagogical work earlier in the education pipeline, or indeed across the general population through citizen-science, given the possibility for such work to support domain-general skills around scientific literacy. This work may help address the growing societal needs to tackle dis- and misinformation, conspiracy theories and evidence manipulation. In addition, this work enables the student, regardless of what career path they may choose, to be a more critical consumer of research, consequently allowing them to develop confidence in challenging outdated dogmas, stereotypes and prejudiced viewpoints. Furthermore, it is also important to appreciate that engagement with open scholarship practices may have unintended negative consequences that our review has not captured. For example, some scholars have raised concerns surrounding the workload that open science adds, and the need for open science discourses to be more compassionate and welcoming (e.g. see [24]). Therefore, we encourage educators to also investigate the potential negative and/or unintended consequences of engagement with open research for students and early-career researchers (see [120] for a useful guide).

## 7. Conclusion

Our literature review has indicated that explicitly embedding open and reproducible scholarship into Higher Education may be associated with student engagement, scientific literacy and attitudes. Furthermore, our review also demonstrated that there remains an imbalance between students' attitudes and students’ behaviours, whereby students often do not see the relevance of open scholarship to their own work, posing a useful area for future follow-up studies and interventions. While there is promising ongoing work in this area, including discussions surrounding the pedagogical value of open scholarship [46] and current Registered Reports that directly and empirically assess the impact of open scholarship on student attitudes [121,122], further empirical research should continue to investigate how embedding open and reproducible science in teaching can affect student outcomes. Beyond our review, there are examples of best practice embedding of open and reproducible scholarship which were not included here, due to the lack of concrete information regarding student outcomes (e.g. [93,97]). Such examples provide a useful framework for integrating open and reproducible science in different levels of the student experience in a range of contexts. These are shared in supplementary information accompanying this manuscript: https://osf.io/4jqbw/. Finally, it is important to stress that the values of open, robust, transparent and rigorous research should be applied to pedagogical research and teaching evaluations, in order to have the best evidence possible to determine the impact of open and reproducible scholarship. In sum: robust research requires robust teaching, and vice versa.

## Data Availability

The data are provided in the electronic supplementary material [123].

## Footnotes

1 ‘Open scholarship’ is more expansive than the term ‘open science’ and includes elements of scientific and methodological rigor, with the addition of being more inclusive than ‘open science’, by extending our knowledge system from the scientific disciplines to all disciplines, including the humanities. In addition, it places focus on aspects of academia that are not typically associated with research such as mentoring, teaching and producing open educational materials [1–4]. In contrast with the open science movement, open scholarship can be used as a tool to aid in the dismantling of the structural barriers that impede accessibility, diversity, equity and inclusion in order to improve science, and includes a humanistic approach by ensuring that voices that have been marginalized such as individuals in the Global South and neurodiverse individuals to be included in the scientific discourse [3,5].

2 We did not conduct a meta-analysis, for two key reasons: (i) many of the studies simply are not suited to meta-analysis (e.g. the review highlighted many qualitative studies and pilot studies) and (ii) the quantitative studies are highly variable in the topics and research questions addressed, and thus do not lend themselves to being pooled in a meta-analysis.

## References

[1] Azevedo F, Liu M, Pennington CR, Pownall M, Evans TR, Parsons S, Elsherif M, Micheli L, Westwood SJ and FORRT. 2022 Towards a culture of open scholarship: the role of pedagogical communities. BMC Res. Notes **15**, 75. (10.1186/s13104-022-05944-1) 35193662 PMC8862562

[2] Azevedo F, Middleton S, Phan JM, Kapp S, Gourdon-Kanhukamwe A, Iley B, Elsherif M, Shaw JJ. 2022 Navigating academia as neurodivergent researchers: promoting neurodiversity within Open Scholarship. APS Observer **35**. See https://www.psychologicalscience.org/observer/gs-navigating-academia-as-neurodivergent-researchers.

[3] Azevedo F et al. 2019 Introducing a Framework for Open and Reproducible Research Training (FORRT). PsyArXiv. (10.31219/osf.io/bnh7p)

[4] Parsons S et al. 2022 A community-sourced glossary of open scholarship terms. Nat. Hum. Behav. **6**, 312-318. (10.1038/s41562-02101269-4) 35190714

[5] Manalili MA et al. 2023 From puzzle to progress: how engaging with neurodiversity can improve cognitive science. Cogn. Sci. **47**, e13255. (10.1111/cogs.13255) 36807910 PMC7616419

[6] Chambers CD, Tzavella L. 2022 The past, present and future of registered reports. Nat. Hum. Behav. **6**, 29-42. (10.1038/s41562-021-01193-7) 34782730

[7] Lindsay DS, Simons DJ, Lilienfeld SO. 2016 Research preregistration 101. Observer **29**. See https://www.psychologicalscience.org/observer/researchpreregistration-101.

[8] Nosek BA et al. 2015 Promoting an open research culture. Science **348**, 1422-1425. (10.1126/science.aab2374) 26113702 PMC4550299

[9] Houtkoop BL, Chambers C, Macleod M, Bishop DV, Nichols TE, Wagenmakers EJ. 2018 Data sharing in psychology: a survey on barriers and preconditions. Adv. Methods Pract. Psychol. Sci. **1**, 70-85. (10.1177/2515245917751886)

[10] Nosek BA, Bar-Anan Y. 2012 Scientific utopia: I. Opening scientific communication. Psychol. Inquiry **23**, 217-243. (10.1080/1047840X.2012.692215)

[11] Delios A et al. 2022 Examining the generalizability of research findings from archival data. Proc. Natl Acad. Sci. USA **119**, e2120377119. (10.1073/pnas.2120377119) 35858443 PMC9335312

[12] Open Science Collaboration. 2015 Estimating the reproducibility of psychological science. Science **349**, 1-10. (10.1126/science.aac4716) 26315443

[13] Tierney W et al. 2020 Creative destruction in science. Org. Behav. Hum. Dec. Process. **161**, 291-309. (10.1016/j.obhdp.2020.07.002)

[14] Tierney W et al. 2021 A creative destruction approach to replication: Implicit work and sex morality across cultures. J. Exp. Soc. Psychol. **93**, 104060. (10.1016/j.jesp.2020.104060)

[15] Farrar BG, Boeckle M, Clayton NS. 2020 Replications in comparative cognition: what should we expect and how can we improve? Anim. Behav. Cogn. **7**, 1-22. (10.26451/abc.07.01.02.2020) 32626823 PMC7334049

[16] Farrar BG et al. 2022 Reporting and interpreting non-significant results in animal cognition research. *PsyArXiv*. (10.31234/osf.io/g9ja2) PMC10008313 36919170

[17] Errington TM, Mathur M, Soderberg CK, Denis A, Perfito N, Iorns E, Nosek BA. 2021 Investigating the replicability of preclinical cancer biology. eLife **10**, e71601. (10.7554/eLife.71601) 34874005 PMC8651293

[18] Camerer CF et al. 2016 Evaluating replicability of laboratory experiments in economics. Science **351**, 1433-1436. (10.1126/science.aaf0918) 26940865

[19] Baum M, Hart A, Elsherif M, Ilchovska ZG, Moreau D, Dokovova M, LaPlume AA, Krautter K, Staal J. 2022 Research without borders: how to identify and overcome potential pitfalls in international large-team online research projects. SAGE Res. Methods Cases. (10.4135/9781529602074)

[20] Munafò MR, Chambers C, Collins A, Fortunato L, Macleod M. 2022 The reproducibility debate is an opportunity, not a crisis. BMC Res. Notes **15**, 1-3. (10.1186/s13104-022-05942-3) 35144667 PMC8832688

[21] Stewart SLK, Pennington CR, da Silva GR, Ballou N, Butler J, Dienes Z, Jay C, Rossit S, Samara A and U. K. Reproducibility Network (UKRN) Local Network Leads. 2022 Reforms to improve reproducibility and quality must be coordinated across the research ecosystem: the view from the UKRN Local Network Leads. BMC Res. Notes **15**, 58. (10.1186/s13104-022-05949-w) 35168675 PMC8845353

[22] Devezer B, Navarro DJ, Vandekerckhove J, Ozge Buzbas E. 2021 The case for formal methodology in scientific reform. R. Soc. Open Sci. **8**, 200805. (10.1098/rsos.200805) 34035933 PMC8101540

[23] Ledgerwood A et al. 2022 The pandemic as a portal: reimaging psychological science as truly open and inclusive. Perspect. Psychol. Sci. **17**, 937-959. (10.1177/17456916211036654) 35235485

[24] Whitaker K, Guest O. 2020 #bropenscience is broken science. The Psychologist **33**, 34-37.

[25] Pownall M et al. 2021 Embedding open and reproducible science into teaching: a bank of lesson plans and resources. Scholarship Teach. Learn. Adv. Online Publication. (10.1037/stl0000307)

[26] Chambers CD. 2019 The seven deadly sins of psychology. Princeton, NJ: Princeton University Press.

[27] Hales AH, Wesselmann ED, Hilgard J. 2019 Improving psychological science through transparency and openness: an overview. Perspect. Behav. Sci. **42**, 13-31. (10.1007/s40614-018-00186-8) 31976419 PMC6701696

[28] Vazire S. 2018 Implications of the credibility revolution for productivity, creativity, and progress. Perspect. Psychol. Sci. **13**, 411-417. (10.1177/1745691617751884) 29961410

[29] Willinsky J. 2006 The access principle: the case for open access to research and scholarship. Cambridge, MA: MIT Press.

[30] * Anglin SM, Edlund JE. 2020 Perceived need for reform in field-wide methods and the teaching of replication, interpretation, and transparency. Psychol. Learn. Teaching **19**, 60-76. (10.1177/1475725719859453)

[31] Button K. 2018 Reboot undergraduate courses for reproducibility. Nature **561**, 287-288. (10.1038/d41586-018-06692-8) 30232437

[32] Nosek et al. 2022. Replicability, robustness, and reproducibility in psychological science. *Annual Review of Psychology* 73, 719–748. (10.1146/annurev-psych-020821-114157) 34665669

[33] Puthillam A, Montilla Doble LJ, Delos Santos JI, Elsherif MM, Steltenpohl CN, Moreau D, Pownall M, Kapoor H. 2022 Guidelines to Improve Internationalization in Psychological Science. *PsyArXiv*. (10.31234/osf.io/2u4h5)

[34] Topor M et al. 2020 An integrative framework for planning and conducting non-intervention, reproducible, and open systematic reviews (NIRO-SR). *MetaArXiv*. (10.31222/osf.io/8gu5z)

[35] Methley AM, Campbell S, Chew-Graham C, McNally R, Cheraghi-Sohi S. 2014 PICO, PICOS and SPIDER: a comparison study of specificity and sensitivity in three search tools for qualitative systematic reviews. BMC Health Serv. Res. **14**, 1. (10.1186/s12913-014-0579-0) 25413154 PMC4310146

[36] Forscher PS, Wagenmakers E, Coles NA, Silan MA, Dutra NB, Basnight-Brown D, IJzerman H. 2020 The benefits, barriers, and risks of Big Team Science. *PsyArXiv*. (10.31234/osf.io/2mdxh) 36190899

[37] * Afolabi F. 2017 First year learning experiences of university undergraduates in the use of open educational resources in online learning. Int. Rev. Res. Open Distrib. Learn. **18**, 1-14. (10.19173/irrodl.v18i7.3167)

[38] * Al Abri MH, Dabbagh N. 2019 Testing the intervention of OER renewable assignments in a college course. Open Praxis **11**, 195-209. (10.5944/openpraxis.11.2.916)

[39] * Altunoglu A. 2017 Initial perceptions of open higher education students with learner management systems. Turkish Online J. Dist. Educ. **18**, 96-104. (10.17718/tojde.328939)

[40] * Andone D, Mihaescu V, Vert S, Ternauciuc A, Vasiu R. 2020 Students as OERs (Open Educational Resources) co-creators. In 2020 IEEE 20th Int. Conf. on Advanced Learning Technologies (ICALT), pp. 34-–38: IEEE.

[41] * Baran E, AlZoubi D. 2020 Affordances, challenges, and impact of open pedagogy: examining students' voices. Dist. Educ. **41**, 230-244. (10.1080/01587919.2020.1757409)

[42] * Becker M. 2020 Qualitative replication as a pedagogical approach to teaching research methods. Polit. Sci. Polit. **53**, 802-806. (10.1017/S1049096520000864)

[43] * Bloom M. 2019 Assessing the impact of ‘Open Pedagogy’ on student skills mastery in first-year composition. Open Praxis **11**, 343-353. (10.5944/openpraxis.11.4.1025)

[44] * Button KS, Chambers, CD, Lawrence N, Munafò MR. 2020. Grassroots training for reproducible science: a consortium-based approach to the empirical dissertation. Psychol. Learn. Teach. **19**, 77-90. (10.1177/1475725719857659)

[45] * Çetinkaya-Rundel M, Ellison V. 2021 A fresh look at introductory data science. J. Stat. Data Sci. Educ. **29**(sup1), S16-S26. (10.1080/10691898.2020.1804497)

[46] * Chopik WJ, Bremner RH, Defever AM, Keller VN. 2018 How (and whether) to teach undergraduates about the replication crisis in psychological science. Teach. Psychol. **45**, 158-163. (10.1177/0098628318762900)

[47] * Davis C, Parmenter L. 2021 Student-staff partnerships at work: epistemic confidence, research-engaged teaching and vocational learning in the transition to higher education. Educ. Action Res. **29**, 292-309. (10.1080/09650792.2020.1792958)

[48] * Hare S, Frye JM, Samuelson BL. 2020 Open pedagogy as an approach to introducing doctoral students to open educational resources and information literacy concepts. Library Trends **69**, 435-468. (10.1353/lib.2020.0041)

[49] * Jekel M, Fiedler S, Allstadt Torras R, Mischkowski D, Dorrough AR, Glöckner A. 2020 How to teach open science principles in the undergraduate curriculum—The Hagen cumulative science project. Psychol. Learn. Teach. **19**, 91-106. (10.1177/1475725719868149)

[50] * Krishna A, Peter SM. 2018 Questionable research practices in student final theses – Prevalence, attitudes, and the role of the supervisor's perceived attitudes. PLoS ONE **13**, e0203470. (10.1371/journal.pone.0203470) 30161249 PMC6117074

[51] * Lin H. 2019 Teaching and learning without a textbook: undergraduate student perceptions of Open Educational Resources. Int. Rev. Res. Open Distrib. Learn. **20**. 10.19173/irrodl.v20i4.4224

[52] * Marwick B, Wang LY, Robinson R, Loiselle H. 2020 How to use replication assignments for teaching integrity in empirical archaeology. Adv. Archaeol. Pract. **8**, 78-86. (10.1017/aap.2019.38)

[53] * Marshall EC, Underwood A. 2019 Writing in the discipline and reproducible methods: a process-oriented approach to teaching empirical undergraduate economics research. J. Econ. Educ. **50**, 17-32. (10.1080/00220485.2018.1551100)

[54] * McCright AM. 2012 Enhancing students' scientific and quantitative literacies through an inquiry-based learning project on climate change. J. Scholarship Teach. Learn. **12**, 86-101.

[55] * Pan SJA. 2021 Taiwanese and American graduate students' misconceptions regarding responsible conduct of research: a cross-national comparison using a two-tier test approach. Sci. Eng. Ethics **27**, 1-23. (10.1007/s11948-021-00297-7) 33765203

[56] * Paviotti G, D'Angelo I, Giaconi C, Cavicchi A. 2020 Open pedagogy practices: a case study in undergraduate education. J. e-Learn. Knowledge Soc. **16**, 1-10. (10.20368/1971-8829/1135321)

[57] * Poronnik P, Moni RW. 2006 The opinion editorial: teaching physiology outside the box. Adv. Physiol. Educ. **30**, 73-82. (10.1152/advan.00075.2005) 16709737

[58] * Pyott L. 2021 Tennis anyone? Teaching experimental design by designing and executing a tennis ball experiment. J. Stat. Data Sci. Educ. **29**, 22-26. (10.1080/10691898.2020.1854638)

[59] * Ryan C. 2020 Students learning as researchers of curriculum in an undergraduate programme. Innovat. Educ. Teach. Int. **57**, 644-654. (10.1080/14703297.2019.167320)

[60] * Sacco DF, Brown M. 2019 Assessing the efficacy of a training intervention to reduce acceptance of questionable research practices in psychology graduate students. J. Emp. Res. Hum. Res. Ethics **14**, 209-218. (10.1177/1556264619840525) 30943835

[61] * Sanchez S, Carter DE, Morey T, Fedorek B. 2021 Student perceptions of an open educational resource for an introduction to criminal justice course. J. Crim. Justice Educ. 1-16. (10.1080/10511253.2021.1986084)

[62] * Sarafoglou A, Hoogeveen S, Matzke D, Wagenmakers EJ. 2019 Teaching good research practices: protocol of a research master course. Psychol. Learn. Teach. **19**, 46-59. (10.1177/1475725719858807)

[63] * Sawchuk D. 2018 The birthday card exercise: replicating research as active learning. Gerontol. Geriatr. Educ. **39**, 481-490. (10.1080/02701960.2016.1247071) 27740894

[64] * Smith LM, Yu F, Schmid KK. 2021 Role of replication research in biostatistics graduate education. J. Stat. Data Sci. Educ. **29**, 95-104. (10.1080/10691898.2020.1844105)

[65] * Steinhardt I. 2020 Learning open science by doing open science. A reflection of a qualitative research project-based seminar. Educ. Inform. **36**, 263-279. (10.3233/EFI-190308)

[66] * Stiemsma LT, Davis SD, Brewster JL. 2020 Analysis of microbial water contamination, soil microbial community structure, and soil respiration in a collaborative first-year students as scholars program (SAS). Front. Microbiol. **11**, 3133. (10.3389/fmicb.2020.590035) PMC7773706 33391206

[67] * Tillinghast B, Fialkowski MK, Draper J. 2020 Exploring aspects of Open Educational Resources through OER-enabled pedagogy. Front. Educ. **5**, 1-14. (10.3389/feduc.2020.00076)

[68] * Toelch U, Ostwald D. 2018 Digital open science—Teaching digital tools for reproducible and transparent research. PLoS Biol. **16**, e2006022. (10.1371/journal.pbio.2006022) 30048447 PMC6095603

[69] * Wang HH, Hong ZR, She HC, Smith TJ, Fielding J, Lin HS. 2022. The role of structured inquiry, open inquiry, and epistemological beliefs in developing secondary students’ scientific and mathematical literacies. Int. J. STEM Educ. **9**, 411-417. (10.1186/s40594-022-00329-z)

[70] * Watson CE, Domizi DP, Clouser SA. 2017 Student and faculty perceptions of OpenStax in high enrollment courses. Int. Rev. Res. Open Distrib. Learn. **18**, 1-18. (10.19173/irrodl.v18i5.2462)

[71] * Werth E, Williams K. 2021 Exploring student perceptions as co-authors of course material. Open Praxis **13**, 53-67. (10.5944/openpraxis.13.1.1187)

[72] OECD. 2006 Measuring student knowledge and skills: the PISA 2000 assessment of Reading, mathematical and scientific literacy. Berlin, Germany: OECD Publishing. See https://www.oecd.org/education/school/programmeforinternationalstudentassessmentpi.

[73] OECD. 2017 PISA 2015 assessment and analytical framework: science, reading, mathematic, financial literacy and collaborative problem solving, revised edition. Berlin, Germany: OECD Publishing.

[74] Tang KS, Williams PJ. 2019 STEM literacy or literacies? Examining the empirical basis of these constructs. Rev. Educ. **7**, 675-697. (10.1002/rev3.3162)

[75] Hubbard K. 2021 Disciplinary literacies in STEM: what do undergraduates read, how do they read it, and can we teach scientific reading more effectively? Higher Educ. Pedagogies **6**, 41-65. (10.1080/23752696.2021.1882326)

[76] American Psychological Association. 2013 APA guidelines for the undergraduate psychology major: version 2.0. Washington, DC: American Psychological Association. 10.1037/a0037562 26866986

[77] American Psychological Association. 2018 Guidelines on core learning goals for master's degree graduates in psychology. Washington, DC: APA. See https://www.apa.org/about/policy/masters-goals-guidelines.pdf.

[78] Blincoe S, Buchert S. 2020 Research preregistration as a teaching and learning tool in undergraduate psychology courses. Psychol. Learn. Teach. **19**, 107-115. (10.1177/1475725719875844)

[79] Pownall M. 2020 Pre-registration in the undergraduate dissertation: a critical discussion. Psychol. Teach. Rev. **26**, 71-76. (10.31234/osf.io/egbcv)

[80] Janz N. 2019 Teaching replication. In Project TIER 2021 Spring Symposium: Instruction in Reproducible Research, *Online, 05 March–21 May 2021*. Project TIER. *See* https://www.projecttier.org/events/2021-spring-symposium-instruction-in-reproducible-research/.

[81] Feldman G. 2022 Replications and extensions of classic findings in judgment and decision making. Open Sci. Framework. (10.17605/OSF.IO/5Z4A8)

[82] Brick C, Fillon A, Yeung SK, Wang M, Lyu H, Ho JYJ, Wong SC, Feldman G. 2021 Self-interest is overestimated: two successful pre-registered replications and extensions of Miller and Ratner (1998). Collabra: Psychology **7**, 23443. (10.1525/collabra.23443)

[83] Chandrashekar SP, Weber J, Chan SY, Cho WY, Chu TCC, Cheng BL, Feldman G. 2021 Accentuation and compatibility: replication and extensions of Shafir (1993) to rethink Choosing versus Rejecting paradigms. Judg. Decision Making **16**, 36-56.

[84] Ziano I, Mok PY, Feldman G. 2021 Replication and extension of Alicke (1985) better-than-average effect for desirable and controllable traits. Soc. Psychol. Pers. Sci. **12**, 1005-1017. (10.1177/1948550620948973)

[85] Feldman G. 2021 Collection of teaching evaluations. (10.17605/OSF.IO/24FJS)

[86] Jarke H et al. 2022 A roadmap to large-scale multi-country replications in psychology. Collabra: Psychology **8**, 57538. (10.1525/collabra.57538)

[87] Field AP. 2014 Skills in mathematics and statistics in psychology and tackling transition. *Higher Education Academy STEM Series*. Sussex, UK: Higher Education Academy.

[88] Bonwell CC, Eison JA. 1991. Active Learning: Creating Excitement in the Classroom. *ERIC Digest*. 1-6. See https://eric.ed.gov/?id=ED336049.

[89] Wagge JR, Brandt MJ, Lazarevic LB, Legate N, Christopherson C, Wiggins B, Grahe JE. 2019 Publishing research with undergraduate students via replication work: the collaborative replications and education project. Front. Psychol. **10**, 1-4. (10.3389/fpsyg.2019.00247) 30814966 PMC6381006

[90] IJzerman HR. 2019 Research tools in social cognition [unpublished course syllabus]. See https://www.dropbox.com/s/z8njk19szkw4u1y/IJzerman%20-%20Research%20Tools%20in%20Social%20Cognition%20Final%202019.docx?dl=0.

[91] Haas HA, Rouse SV. 2022 Learning from mistakes: teaching students about errata, corrigenda, and nonretraction corrections to the research literature. Scholarship Teach. Learn. Psychol. **8**, 58-69. (10.1037/stl0000216)

[92] Frank MC, Saxe R. 2012 Teaching replication. Perspect. Psychol. Sci. **7**, 600-604. (10.1177/1745691612460686) 26168118

[93] Kathawalla UK, Silverstein P, Syed M. 2021 Easing into open science: a guide for graduate students and their advisors. Collabra: Psychology **7**, 18684. (10.1525/collabra.18684)

[94] Nurse AM, Staiger T. 2019 Teaching data reproducibility through service learning. Teach. Sociol. **47**, 350-357. (10.1177/0092055X19860577)

[95] Kahu ER, Stephens C, Leach L, Zepke N. 2013 The engagement of mature distance students. Higher Educ. Res. Dev. **32**, 791-804. (10.1080/07294360.2013.777036)

[96] Groccia JE. 2018 What is student engagement? New Dir. Teach. Learn. **2018**, 11-20. (10.1002/tl.20287)

[97] McAleer P. 2021 Creating a curriculum centered on reproducible research for the psychologists of the future. In Project TIER 2021 Spring Symp.: Instruction in Reproducible Research, Online, 5 March–21 May 2021. Project TIER. See https://www.projecttier.org/events/Phil-McAleer-Creating-a-curriculum-centered/.

[98] Barr D. 2016 No more excuses: R is better than SPSS for psychology undergrads, and students agree. DataHowler. See https://datahowler.wordpress.com/2016/09/10/no-moreexcuses-r-is-better-than-spss-for-psychology-undergrads-and-students-agree/.

[99] Bangera G, Brownell SE. 2014 Course-based undergraduate research experiences can make scientific research more inclusive. CBE—Life Sci. Educ. **13**, 602-606. (10.1187/cbe.14-06-0099) 25452483 PMC4255347

[100] Clark et al. 2020 Development, implementation and importance of an undergraduate peer research consultant program at the University of North Dakota’s Chester Fritz Library. *Reference Services Review* **48**, 579–600.

[101] Pennington CR, Jones AJ, Tzavella L, Chambers CD, Button KS. 2022 Beyond online participant crowdsourcing: the benefits and opportunities of big team addiction science. Exp. Clin. Psychopharmacol. Adv. Online Publication. (10.1037/pha0000541) 35025584

[102] Button et al. 2018 Reboot undergraduate courses for reproducibility. *Nature* **561**, 287–288. 10.1038/d41586-018-06692-8 30232437

[103] Lindshield BL, Adhikari K. 2013 Online and campus college students like using an open educational resource instead of a traditional textbook. J. Online Learn. Teach. **9**, 26-38.

[104] Zapata GC. 2020 Sprinting to the finish line: the benefits and challenges of book sprints in OER faculty-graduate student collaborations. Int. Rev. Res. Open Distrib. Learn. **21**, 1-17. (10.19173/irrodl.v21i2.4607)

[105] Ajzen I, Fishbein M. 2005 The influence of attitudes on behavior. In The handbook of attitudes (eds D Albarracin, BT Johnson, MP Zanna), pp. 173-221. New York, NY: Erlbaum.

[106] Hardwicke TE, Thibault RT, Kosie JE, Wallach JD, Kidwell MC, Ioannidis JP. 2022 Estimating the prevalence of transparency and reproducibility-related research practices in psychology (2014–2017). Perspect. Psychol. Sci. **17**, 239-251. (10.1177/1745691620979806) 33682488 PMC8785283

[107] Serghiou S, Contopoulos-Ioannidis DG, Boyack KW, Riedel N, Wallach JD, Ioannidis JP. 2021 Assessment of transparency indicators across the biomedical literature: how open is open? PLoS Biol. **19**, 1-26. (10.1371/journal.pbio.3001107) PMC7951980 33647013

[108] Hanna S, Pither J, Vis-Dunbar M. 2021 Implementation of an open science instruction program for undergraduates. Data Intelligence **3**, 150-161. (10.1162/dint_a_00086)

[109] Truan N, Dressel D. 2021 Doing open science in a research-based seminar: students positioning towards openness in higher education. HAL SHS Hum. Soc. Sci. **23**, 1-19. See https://halshs.archives-ouvertes.fr/halshs-03395171.

[110] Olsen J, Mosen J, Voracek M, Kirchler E. 2019 Research practices and statistical reporting quality in 250 economic psychology master's theses: a meta-research investigation. R. Soc. Open Sci. **6**, 190738. (10.1098/rsos.190738) 31903199 PMC6936276

[111] Giuliano T, Skorinko JLM, Fallon M. 2019 Engaging undergraduates in publishable research: best practices. Front. Psychol. **1878**, 1-6. (10.3389/fpsyg.2019.01878) PMC6719508 31507477

[112] Rosenthal R. 1979 The file drawer problem and tolerance for null results. Psychol. Bull. **86**, 638-641. (10.1037/0033-2909.86.3.638)

[113] Poldrack RA et al. 2017 Scanning the horizon: towards transparent and reproducible neuroimaging research. Nat. Rev. Neurosci. **18**, 115-126. (10.1038/nrn.2016.167) 28053326 PMC6910649

[114] Simonsohn U, Nelson LD, Simmons JP. 2014 p-Curve and effect size: correcting for publication bias using only significant results. Perspect. Psychol. Sci. **9**, 666-681. (10.1177/1745691614553988) 26186117

[115] Burgstahler SE, Cory RC. 2010 Universal design in higher education: from principles to practice. Cambridge, MA: Harvard Education Press.

[116] Gourdon-Kanhukamwe A et al. 2023 Promoting participatory research for neurodiverse populations through Open Scholarship practice. BPS Cogn. Psychol. Bullet. **8**, 23. (10.53841/bpscog.2023.1.8.23)

[117] Elsherif MM et al. 2022 Bridging Neurodiversity and Open Scholarship: How Shared Values Can Guide Best Practices for Research Integrity, Social Justice, and Principled Education. *MetaArXiv*. (10.31222/osf.io/k7a9p)

[118] Nordmann E, Clark A, Spaeth E, MacKay JR. 2021 Lights, camera, active! Appreciation of active learning predicts positive attitudes towards lecture capture. Higher Educ. **83**, 481-502. (10.1007/s10734-020-00674-4) PMC7812336 33487670

[119] Nightingale KP, Anderson V, Onens S, Fazil Q, Davies H. 2019 Developing the inclusive curriculum: is supplementary lecture recording an effective approach in supporting students with Specific Learning Difficulties (SpLDs)? Comput. Educ. **130**, 13-25. (10.1016/j.compedu.2018.11.006)

[120] Kowalczyk OS, Lautarescu A, Blok E, Dall'Aglio L, Westwood SJ. 2022 What senior academics can do to support reproducible and open research: a short, three-step guide. BMC Res. Notes **15**, Article 116. (10.1186/s13104-02205999-0) PMC8938725 35317865

[121] Jarke H, Jakob L, Bojanić L, Garcia-Garzon E, Mareva S, Mutak A, Gjorgjiovska J. 2022 Registered report: how open do you want your science? An international investigation into knowledge and attitudes of psychology students. PLoS ONE **17**, e0261260. (10.1371/journal.pone.0261260) 35226677 PMC8884512

[122] Pownall M, Pennington CR, Norris E, Clark K. 2021 Evaluating the pedagogical effectiveness of study preregistration in the undergraduate dissertation: a registered report. Open Sci. Framework. See https://osf.io/5qshg/.

[123] Pownall M et al. 2023 Teaching open and reproducible scholarship: a critical review of the evidence base for current pedagogical methods and their outcomes. Figshare. (10.6084/m9.figshare.c.6641489) PMC10189598 37206965

